# The core genome of the anaerobic oral pathogenic bacterium *Porphyromonas gingivalis*

**DOI:** 10.1186/1471-2180-10-252

**Published:** 2010-09-29

**Authors:** Jorg Brunner, Floyd RA Wittink, Martijs J Jonker, Mark de Jong, Timo M Breit, Marja L Laine, Johannes J de Soet, Wim Crielaard

**Affiliations:** 1 Department of Oral Microbiology, Academic Centre for Dentistry Amsterdam, University of Amsterdam and Free University Amsterdam, Amsterdam, The Netherlands; 2 Microarray Department and Integrative Bioinformatics Unit, Swammerdam Institute for Life Sciences, University of Amsterdam, Amsterdam, The Netherlands

## Abstract

**Background:**

The Gram negative anaerobic bacterium *Porphyromonas gingivalis *has long been recognized as a causative agent of periodontitis. Periodontitis is a chronic infectious disease of the tooth supporting tissues eventually leading to tooth-loss. Capsular polysaccharide (CPS) of *P. gingivalis *has been shown to be an important virulence determinant. Seven capsular serotypes have been described. Here, we used micro-array based comparative genomic hybridization analysis (CGH) to analyze a representative of each of the capsular serotypes and a non-encapsulated strain against the highly virulent and sequenced W83 strain. We defined absent calls using *Arabidopsis thaliana *negative control probes, with the aim to distinguish between aberrations due to mutations and gene gain/loss.

**Results:**

Our analyses allowed us to call aberrant genes, absent genes and divergent regions in each of the test strains. A conserved core *P. gingivalis *genome was described, which consists of 80% of the analyzed genes from the sequenced W83 strain. The percentage of aberrant genes between the test strains and control strain W83 was 8.2% to 13.7%. Among the aberrant genes many CPS biosynthesis genes were found. Most other virulence related genes could be found in the conserved core genome. Comparing highly virulent strains with less virulent strains indicates that *hmuS, *a putative CobN/Mg chelatase involved in heme uptake, may be a more relevant virulence determinant than previously expected. Furthermore, the description of the 39 W83-specific genes could give more insight in why this strain is more virulent than others.

**Conclusion:**

Analyses of the genetic content of the *P. gingivalis *capsular serotypes allowed the description of a *P. gingivalis *core genome. The high resolution data from three types of analysis of triplicate hybridization experiments may explain the higher divergence between *P. gingivalis *strains than previously recognized.

## Background

Periodontitis is a chronic destructive infectious disease of the tooth-supporting tissues. It is one of the most prevalent infectious diseases in the world. With percentages of moderate disease ranging from just below 20% in an age group of 30 to 40 year-olds in Swedish and Norwegian studies to even up to 38% of severe cases in the United States in an on average 75 year-old male population [1-3]. Besides high prevalence also links to systemic diseases have been described. Periodontitis has been associated with, amongst others, cardiovascular diseases, diabetes mellitus and rheumatoid arthritis [4-7].

Periodontitis leads to loss of sound teeth as supporting bone and connective tissue are slowly degraded as a result of an exaggerated host immune response triggered against a polymicrobial biofilm [8].

In the oral cavity around 7000 species can be detected, in subgingival and supragingival biofilm/plaque over 400 bacterial species are present [9-11]. Many disease-related bacterial species in the subgingival plaque have been shown to be Gram-negative anaerobes. Among them, *Porphyromonas gingivalis *a black-pigmented bacterium from the phylum *Bacteroidetes *is a major causative agent in periodontal disease [12].

Interaction with other bacteria residing in the periodontal pocket is important to sustain the infectious biofilm. One of the structures involved in the inter-species adherence is the capsular polysaccharide (CPS) of *P. gingivalis *[13]. CPS has been described as a virulence factor of various pathogenic bacteria, mainly as being involved in evasion of the host immune system [14-16]. In *P. gingivalis *encapsulated strains have been shown to be more resistant to serum killing and phagocytosis. The explanation for this increased resistance compared to the non-encapsulated strains may be the increased hydrophilicity and the lower induction of the alternative complement pathway [17].

Encapsulated *P. gingivalis *strains have also been shown to be more virulent than non-encapsulated strains in the mouse infection model [18]. To date, six capsular serotypes (K1-K6) have been described [19,20] and a seventh serotype (K7) has been suggested by R. E. Schifferle (personal communication). In a mouse subcutaneous infection model several strains of each of the serotypes have been shown to be highly virulent [18]. The variation of virulence within serotypes shows that besides CPS there have to be more virulence factors of importance in *P. gingivalis*. Many of its virulence factors have been studied in the last decades including fimbriae, hemagglutinins, lipopolysaccharide (LPS), outer membrane proteins (OMPs) and an extremely wide variety of proteinases. High quality reviews have been published on the wide variety of *P. gingivalis *virulence factors [21-23].

Using comparative whole-genome hybridization analysis of the encapsulated W83 strain and the non-encapsulated ATCC33277 a CPS biosynthesis locus had been found, after which a knock-out study has proven that the CPS locus was functional [24,25]. K1 CPS from W83 has been shown to induce a stronger chemokine response than CPS from the other serotypes in murine macrophages [26]. Recent work in our group, however, has shown that an isogenic W83 mutant lacking CPS triggers a higher pro-inflammatory immune response in human gingival fibroblasts than strain W83 carrying K1 CPS [27]. The exact roles of CPS in *P. gingivalis *are still unclear, but reducing the host immune response is certainly one of them.

In the latest years an increasing number of genomes have been sequenced paving the path for genomics-based approaches. For *P. gingivalis *genome sequences of the virulent strain W83 and the less-virulent strain ATCC33277 have become available [28,29]. Comparative genomic hybridization (CGH) analysis using microarrays of these well-described bacterial strains could yield new insights in the virulence mechanisms of *P. gingivalis*. A recent study reported on the CGH analysis of several *P. gingivalis *strains to describe the genetic variety among them [30].

In this study we analyzed the genetic contents of representative strains of each of the seven capsular serotypes (Table 1): W83 (K1), HG184 (K2), ATCC53977 (K3), ATCC49417 (K4), HG1690 (K5), HG1691 (K6), 34-4 (K7). We also included the non-encapsulated strain FDC381 (K-) in the CGH analysis to compare with each of the encapsulated strains. Strain FDC381 does however express a non-CPS anionic extracellular polysaccharide as do the other strains [31]. The strains were classified into three virulence levels as determined by using a subcutaneous mouse infection model [18,32]. Although not an optimal measure for the ability to cause periodontitis, this classification has long been used [33] and proven useful in studying virulence determinants [34-37].

**Table 1:** *P. gingivalis *strains used in this study

Strain	Capsular serotype	Origin	**Virulence**^**c**^
W83^a^	K1	Clinical specimen	High
HG184	K2	Periodontitis patient	Medium
HG1025	K3	Periodontitis patient with diabetes mellitus	High
ATCC49417	K4	Advanced adult periodontitis patient	High
HG1690	K5	37-year-old male periodontitis patient	High
HG1691	K6	28-year-old female periodontitis patient	Medium
34-4	K7	Severe periodontitis patient	Low
FDC381^b^	K-	Adult periodontitis patient	Low

^a ^A kind gift of H. N. Shah (NCTC, London, UK)

^b ^A kind gift of S. S. Socransky (The Forsyth Institute, Boston, MA, USA)

^c ^As determined in a subcutaneous mouse infection model [18,32]

Triplicate hybridization experiments and three types of analysis, 1) aberrant gene calling, 2) breakpoint analysis and 3) absent gene calling, have been performed for optimal use of the new genetic information. The careful design of the experiment and the thorough analysis of the data lead to a high resolution data set, yielding more detailed information on the genetic differences between strains than has been shown before. In this study we initiate the description of a core-gene set of *P. gingivalis *allowing a more focused search for potential important virulence factors.

## Results and discussion

### Microarray performance and data interpretation

The *P. gingivalis *version 1 microarray from the PFGRC used in this study has been used in several studies before [30,38] and consists of 1907 probes and 500 negative control probes (*Arabidopsis thaliana*) printed in four replicates. The microarray was designed to cover all non-redundant coding sequences (CDSs) of the *P. gingivalis *W83 genome. Before our study all probes were analyzed for their unique- and perfect matching with the genome, as downloaded from the NCBI, using BLAST. Twenty-nine of the 1907 probes of the microarray gave non-specific hits, mostly related to transposases (Table 2). These probes were excluded from further analyses together with four probes that were not in use anymore annotated "obsolete" by the manufacturer, so that 1874 probes remained. The comparison of each test strain to W83 using this array gives insights into described virulence associated genes. A limitation of the method, however, is that genes from the variable gene pool from other strains will not be detected.

**Table 2:** Probes excluded from analysis due to redundancy

GeneID	Annotated function
*PG2152*	DNA-binding protein, histone-like family
*PG0261*	ISPg3, transposase
*PG0943*	ISPg5, transposase Orf2
*PG1420*	ISPg5, transposase Orf2
*PG1444*	hypothetical protein
*PG1261*	ISPg4, transposase
*PG1276*	DNA-binding protein, histone-like family
*PG1670*	hypothetical protein
*PG1451*	conserved hypothetical protein
*PG2128*	ISPg5, transposase Orf2
*PG1449*	conserved hypothetical protein
*PG1453*	Integrase
*PG1267*	hypothetical protein
*PG1350*	ISPg2, transposase
*PG0827*	MATE efflux family protein
*PG1669*	hypothetical protein
*PG1448*	ISPg1, transposase
*PG1709*	ISPg5, transposase Orf1
*PG1454*	Integrase
*PG1332*	NAD(P) transhydrogenase, beta subunit
*PG1452*	lipoprotein, putative
*PG1384*	ISPg1, transposase, authentic frameshift
*PG1244*	ISPg1, transposase
*PG1447*	transcriptional regulator, AraC family
*PG1450*	conserved hypothetical protein
*PG1445*	rteC protein, truncation
*PG1671*	hypothetical protein
*PG0487*	ISPg4, transposase
*PG0760*	ISPg1, transposase, authentic frameshift

**Figure 1:**
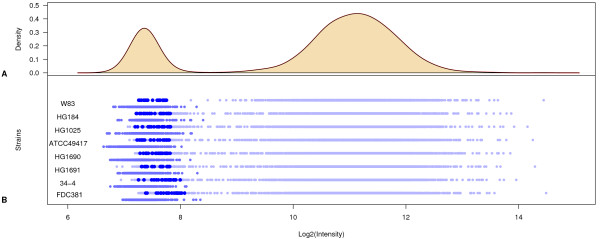
**Hybridization signals of *P. gingivalis *strains - dead probes**. A. The total intensity distribution of probe signals of W83 DNA hybridized to the W83 array. The density peak around 7.5 contains the negative controls (empty spots and *A. thaliana *probes). The peak around 12 should contain all present genes in strain W83. B Probe signal intensities of each *P. gingivalis *test strain are represented in light blue dots; medium blue dots, slightly below that, symbolize *A. thaliana *negative control genes. Dark blue dots represent *P gingivalis *probes, which show the same low intensity as the negative control probes. These 22 probes are called dead probes as they do not give any significant hybridization signal.

Data were normalized and technical and biological replicates were collapsed as described in the Materials and Methods. Detailed analysis of the probe intensities indicated that 22 probes gave systematically low intensity values for strain W83 as well as for all the other strains. The intensity levels were at the same low levels as the intensity levels of the negative control probes (Figure 1). These probes were labeled as "dead probes" and excluded from the results (Table 3). Our data do not explain why dead probes have occurred in our experiments, but the consistent low signal for these probes suggests that the sequencing information used for designing these probes was imperfect.

**Table 3:** Dead probes excluded from the results due to low hybridization signals

GeneID	Annotated function
*PG0222*	DNA-binding protein, histone-like family
*PG0375*	ribosomal protein L13
*PG0498*	autoinducer-2 production protein LuxS
*PG0786*	hypothetical protein
*PG0809*	hypothetical protein
*PG0855*	hypothetical protein
*PG0880*	bacterioferritin comigratory protein
*PG0979*	hypothetical protein
*PG0994*	hypothetical protein
*PG1234*	hypothetical protein
*PG1257*	hypothetical protein
*PG1335*	membrane protein, putative
*PG1357*	hypothetical protein
*PG1412*	ISPg2, transposase, truncation
*PG1617*	hypothetical protein
*PG1660*	RNA polymerase sigma-70 factor, ECF subfamily
*PG1742*	hypothetical protein
*PG1866*	hypothetical protein
*PG1869*	hypothetical protein
*PG1987*	CRISPR-associated protein, TM1794 family
*PG2019*	hypothetical protein
*PG2087*	conserved hypothetical protein

In order to maximize the mining of the genomic information, we subjected the data to three complementary analyses: 1) analysis for aberrations as detected by individual probes, 2) analysis for breakpoints, and 3) analysis for genomic loss. The rationale behind the three analyses is as follows. The probed genomic sites are on average 1250 bp apart from each other (median was 1018), which was not considered to be a high interrogation density. We therefore decided to analyze each probe individually for indication that the genomic site interrogated is aberrant from W83. Deviations from W83 that were detected with a false discovery rate corrected p-value (FDR) < 0.05 were considered significant. This aberrance could have occurred due to mutations or loss (or due to W83 gain), and this was regarded as point-variability between the strains. Nevertheless, if several neighboring probes indicate aberrations, then this may indicate highly variable regions due to mutations or loss. Hence, a breakpoint analysis was executed to quantitatively specify such regions. Finally, we used the negative controls to define absent calls with the aim to distinguish whether an aberration was found more likely due to mutation or loss. If the probes that indicated aberrations in the first analysis also showed the same intensities as the negative controls with FDR corrected p-value < 0.01 (see M&M), the genomic site was considered as mutated, and otherwise it was considered as lost. This last analysis enhanced our interpretation of the data and the definition of the core genome.

### *P. gingivalis *core genome

Research on microbial pathogens is mostly performed to unravel mechanisms of virulence in order to design effective treatments. Virulence mechanisms present in all strains of a species are especially attractive. The description of a core set of genes present in a species is thus a key step for better understanding. From an analysis of eight *P. gingivalis *strains we found that 1476 genes were non-aberrantly present in each of the strains and 2 hypothetical genes were called absent but non-aberrant (Additional file 1). The conserved core genes make up 80% of all genes included in this study. Hence, 20% (374) of all genes of W83 were aberrant in at least one of the strains. Core genomes from several bacterial species have been described [39-45]. The fraction of a bacterial genome that consists of core genes depends highly on the amount of strains included to describe the core genome [43]. The more strains are used, the smaller the core genome will be. As such, the very well studied *Escherichia coli *core genome makes up only 46% of the average *E. coli *genome. Other bacterial species, including Gram positives and Gram negatives, have been found to have a core genome which covers 52% to 85% of a genome [39-45]. The 80% of W83 genes which are part of the conserved core genome can therefore be understood. It must be clear though that the core genome of *P. gingivalis *as described here must be seen as a first step. The core genome will be found to be smaller as more genetic information on different *P. gingivalis *strains will become available.

**Figure 2:**
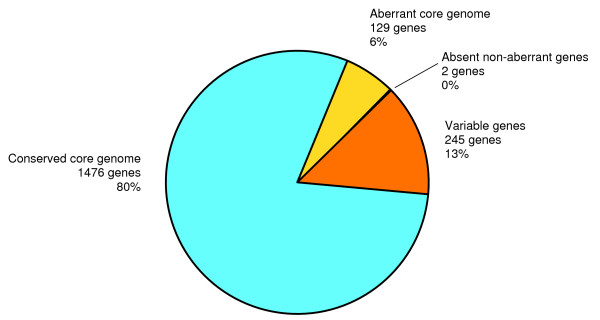
***P. gingivalis *core genome**. Pie diagram representing all probes included in the results divided into pieces representing the conserved core genome, aberrant core genome and the variable genes. The percentages show the proportions of the total of functional probes. 80% of the strain W83 genes is present and conserved among the test strains. 6% of the W83 genes is present but aberrant and 13% of the genes is absent in at least one of the test strains. Two probes with very low signals were found as non-aberrant but absent.

We could distinguish two gene sets in the aberrant set, namely the present and absent genes (Figure 2). Using aberrance and absent call analysis we were thus able to describe the *P. gingivalis *core genome in two ways. Aberrance represents mutations within the probe sequence, whereas absent calls represents the total absence of the probe sequence interpreted as gene absence. The fully conserved *P. gingivalis *core genome is comprised of 1476 genes. The variable core genome is comprised of a total of 1605 genes, which are aberrant, but called present (Figure 2). In the further analyses the conserved core genome was taken as the core genome.

Combining our findings on the core genome with a study describing 1490 conserved CDSs when comparing the genome sequences of W83 and ATCC33277 [28], makes it tempting to speculate that the core genome as described here may already be close to its final size. An analysis combining the conserved CDSs from that study with our 1476 conserved core genes showed that when strain ATCC33277 is included the core genome size decreased to 1384 genes.

**Figure 3:**
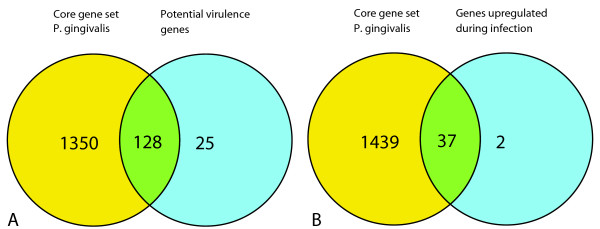
**Virulence associated genes in the conserved core genome of *P. gingivalis***. A. 153 potential virulence genes from the genome annotation of W83 combined with the conserved core genome of *P. gingivalis *[29]. B 39 genes known to be up-regulated during infection combined with the conserved core genome of *P. gingivalis *[46,47]. The number in the overlapping part of the circles is the number of potential virulence associated genes that was found in the conserved core genome of *P. gingivalis.*

The conserved core gene set was analyzed for the presence of virulence genes. When it was analyzed for the presence of the 153 potentially virulence associated gene set originating from when the genome sequence of W83 became available (selected by presence of a signal peptide, or transmembrane domains or by homology to previously described virulence agents) [29], it was found that 128 genes were present in all strains (Figure 3A). Only 25 genes were aberrant in at least one strain, among which 9 usual suspects from the CPS locus, but also four hemagglutinins.

Another virulence gene set was also tested for presence in the conserved core gene set of *P. gingivalis*. The set was composed of genes shown to be up-regulated in infection experiments [46,47]. Genes up-regulated in an *in vitro *human epithelial cell infection experiment were combined with a gene set *in vivo *up-regulated on protein level in a mouse subcutanuous chamber experiment to make a set of 39 virulence genes. The former experiment was chosen as an early response gene set, whereas the latter includes genes involved in sustaining an infection *in vivo*. 37 of the 39 virulence genes were present among the core gene set (Figure 3B). The two genes that were not in the core gene set were a thiol protease (*PG1055*) [48] and *tetR *a transcription regulator (*PG1240*). The thiol protease is aberrant in each strain except for strain ATCC49417, from the 16S-23S ISR heteroduplex type that together with the type of strain W83 has the highest association with disease [49]. This is another indication that this thiol protease may be an important determinant in virulence of *P. gingivalis*. Transcription regulator *tetR *was only found to be aberrant in strain FDC381, which is the least virulent and the only non-encapsulated strain [18,32].

The analysis of the core gene set shows the presence of almost all virulence related genes. The genes that are not present in the core genome may be determinants of the differences in virulence found between the strains.

### Strain divergence

The divergences of the test strains were determined by the percentage of aberrant CDSs from the total number of 1874 CDSs included in this study. We found 8.2% to 13.7% of aberrant genes per strain, with ATCC49417 having the lowest and FDC381 having the highest percentage of aberrant genes (Table 4). These percentages of aberrant genes are higher than the 7% of aberrant genes from a previous genomic hybridization study on strain ATCC33277, a close relative of strain FDC381 [25]. From the 64 highly aberrant genes in ATCC33277 41 genes were included in our study from which 33 were in the aberrant gene list of strain FDC381. A recent study reported even lower percentages 0-5.1% of divergence between *P. gingivalis *strains [30]. Although they used the same arrays and also used some identical strains the differences between our data sets were substantial. We detect a much higher number of aberrant genes probably because of higher resolution due to the use of three arrays per strain. We also excluded a set of 55 genes before the analyses (see above) which further elevated the percentages found in this study.

**Table 4:** Aberrant and absent CDSs of *P. gingivalis *strains

Strain	Aberrant CDSs	% aberrant	Absent CDSs	% absent
HG184	213	11,4	133	7,8
HG1025	214	11,4	135	7,8
ATCC49417	153	8,2	88	4,7
HG1690	187	10,0	107	5,7
HG1691	227	12,1	158	8,5
34-4	207	11,0	126	6,8
FDC381	256	13,7	195	10,5

### Proteases

*P. gingivalis *is known to have a vast arsenal of proteases. The main function of these enzymes is to provide peptides for growth. These peptides can be derived from host-proteins, involved in defence against pathogens, thereby potentially disrupting the host immune response. Other proteases degrade collagen, thereby weakening the tooth-supporting tissues. Proteases have therefore been regarded as important virulence factors. A selection of 64 proteases/peptidases was made by text searches in the *P. gingivalis *W83 genome annotation combined with peptidases found in the MEROPS *P. gingivalis *peptidase database [50] (http://merops.sanger.ac.uk/index.shtml). This selection was analyzed for presence in the test strains. From the analysis it was clear that most proteases, 58 in total, belong to the core gene set of *P. gingivalis*. From the 6 non-core protease genes (Table 5) *tpr *was already mentioned earlier. The gene *prtC*, a collagenase, was found to be aberrant only in three strains with medium/low virulence in a subcutaneous mouse model. Interestingly, in early studies on *P. gingivalis *virulence one of the discriminatory factors between virulent and avirulent strains was described to be collagenase activity, which was found to be low in avirulent strains [51]. Another non-core protease gene is the well-described *rgpA*, an arg-gingipain which has regularly been described as one of the most important virulence factors of *P. gingivalis *[52,53]. *RgpA *is aberrant in the highly virulent strain ATCC53977. This finding is however in line with a murine periodontitis model study in which *rgpA *was found to be not important in virulence using *P. gingivalis *knockouts [34]. From the present study, however, no hard conclusion should be drawn as no functional changes have been explored.

**Table 5:** Non-core protease genes of *P. gingivalis*

GeneID	Annotation	Remark
*PG0317*	peptidase, M49 family	Aberrant only in 34-4
*PG1055*	thiol protease	Non-aberrant only in W83 and ATCC49417 (absent in FDC381)
*PG1542*	collagenase	Aberrant in HG1691, 34-4 and FDC381
*PG2024*	hemagglutinin protein HagE	Aberrant and absent only in HG1025
*PG2115*	protease PrtT, degenerate	Non-aberrant only in W83
*PG2185*	transporter, putative	Aberrant in HG184, HG1025 and FDC381

### The capsular polysaccharide biosynthesis locus

**Figure 4:**
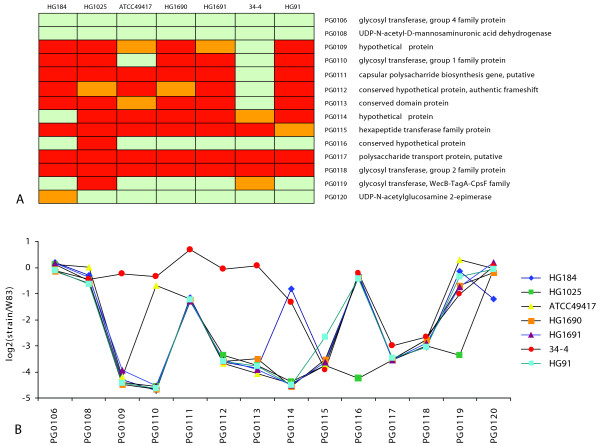
**CPS biosynthesis locus diversity**. A. Heat map showing presence (green), aberrance (orange) and absence (red) of each gene in each test strain, showing the variation within the CPS biosynthesis locus. The CPS locus of the serotype K7 strain 34-4 shows the highest similarity with the K1 serotype strain W83. B. For each probe in the CPS biosynthesis locus and for each test strain a log-ratio value compared to strain W83 is depicted by a data point, supporting the heat map results as shown in figure 4A.

Analysis of the CPS biosynthesis locus [24] of the test strains revealed a high variation as seen in Figure 4A showing the hybridization log-ratios against W83. Our interpretation of the log-ratios depicted as a heat map showing presence, aberrance and absence of each of the CPS-locus genes is shown in Figure 4B. Only *PG0106 *and *PG0108 *show no divergence in any strain and are thus among the core gene set as described earlier. The other genes in the locus show at least some aberrance. *PG0117 *and *PG0118 *are called absent in each test strain as concluded from our hybridization experiments. This supports the choice of these genes to design a K1-specific PCR for serotyping in our group [54]. All test strains are found to be aberrant for at least 8 genes, except strain 34-4 (K7) which only shows aberrance in 5 genes. These findings may suggest that the different capsular serotypes can be highly variable in structure and that K7 CPS may share more common elements with the K1 type of CPS than the other test strains.

### Highly variable regions

**Figure 5:**
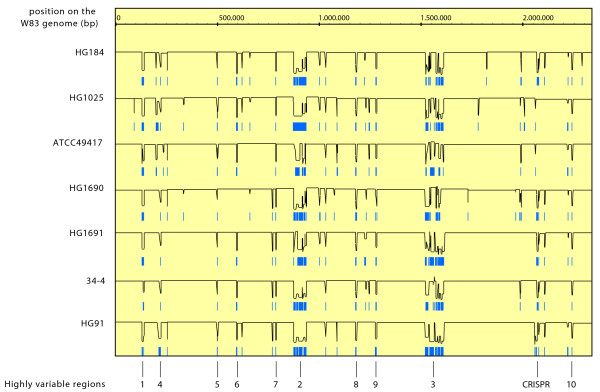
**Highly variable regions of *P. gingivalis***. Breakpoint analysis of test strains describing potential lacking genomic regions as positioned on the W83 genome sequence. Black lines depict breakpoint data. As long as the line is flat there is low variability of the test strain compared to W83. Dips in the line indicate variability. Blue lines/rectangles below depict potential absent regions. At the top the probe positions are given as described in the W83 genome [29]. The numbers at the bottom label the 10 highly variable regions in each strain which are explained in the text. CRISPR represents a region of interest with CRISPR associated genes as described in the text.

An analysis was performed to calculate the chance that certain genetic regions of the W83 genome are missing in the test strains included in the hybridization experiments. This was done using breakpoint analysis, which takes the divergence of neighbouring genes into account. In this analysis 10 highly variable regions were found (Figure 5). Three regions, regions 1, 2 and 3, have already been reported earlier based on aberrance in strain ATCC33277 [25] (Table 6), but only a function for the CPS biosynthesis locus has been described. The function of the other two may be pathogenicity islands, although no prove has been reported yet. Region 4 which includes *ragA *and *ragB *is in addition to W83 only present in strain ATCC49417. Both strains are representatives of the 16S-23S ISR heteroduplex types that have the strongest association with disease. The other strains lack this region. This region has also been described as disease related directly by PCR of subgingival samples [55]. Region 5 includes *pgaA*, which also has been described as a virulence determinant [56]. The other highly variable regions may be involved in virulence, but too little is known to speculate on the functions.

**Table 6:** Highly variable *P. gingivalis *genomic regions

Variable region	Location	Gene content of the region
Region 1	*PG0109-PG0118*	Capsular polysaccharide biosynthesis locus [27,28]
Region 2	*PG0814-PG0875*	Potential pathogenicity island [28]. Many DNA mobilization proteins
Region 3	*PG1435-PG1533*	Potential pathogenicity island [28]. Many transposon related genes.
Region 4	*PG0185-PG0187*	Virulence associated ragA-ragB locus [46] highly variable in strains other than W83 and ATCC49417
Region 5	*PG0456-PG0461*	PHP domain protein, transposases
Region 6	*PG0542-PG0546*	transcriptional regulator, type 1 restriction modification gene
Region 7	*PG0741-PG0742*	PgaA and hypothetical protein
Region 8	*PG1107-PG1113*	Integrase/mobilization, hypothetical proteins
Region 9	*PG1200-PG1206*	Transcriptional regulator, DNA binding protein, hypothetical proteins
Region 10	*PG2134-PG2136*	Lipoproteins, hypothetical proteins

Another region that was found to be interesting in this analysis is region *PG1981*-*PG1986 *which is comprised of clustered regularly interspaced short palindromic repeat (CRISPR) associated genes (CAS) [57]. Together with CRISPRs, located directly downstream of *PG1981*, these types of genes have been described as the immune system of bacteria against foreign DNA, *e.g. *plasmids and viruses. Recently they also have been described as a useful tool in epidemiology [58]. Variation is expected to be high in these regions as they encompass exogenous DNA sequence fragments from infection events that happened to the strain or its ancestors. Here variation within the CAS genes is evident, but not as high as the other regions mentioned in this section.

### W83-specific genes

Strain W83 has been described as a highly virulent strain. What makes this strain special is however not specifically known. The purified CPS of W83 has been shown to induce a higher immune response than other types of CPS [26]. Removal of the capsular structure, by genetic interruption of CPS-biosynthesis, however resulted in a much higher immune response when infecting fibroblasts with viable *P. gingivalis *[27]. What this means for virulence in a mouse model has not yet been addressed. With the data presented here a more detailed study is possible to find specific traits that make W83 different. A list of all genes that are aberrant in each of the test strains and absent in each of the test strains is presented (see Additional file 2). Among the 65 genes that were aberrant in all test strains there were 10 DNA mobilization genes, 5 DNA modification genes, 3 CPS biosynthesis genes, 2 carbohydrate metabolism genes, 2 transcriptional regulator genes, 2 lipoprotein genes and 36 (conserved) hypothetical protein genes. From this gene set 39 genes were W83-specific as they were absent in each of the test strains. In this way the *prtT *protease gene and a fimbrillin gene (*fimA*) were found to be aberrant in all test strains, but not W83-specific as they were present in one or more test strains. The results for *fimA *support the findings that the gene is widely distributed, but variable at the probe locus among *P. gingivalis *strains. Many of the genes found in this analysis are located within the highly variable regions described in earlier publications using whole-genome analysis. The existence of those regions were supported by data comparing the genome sequences of *P. gingivalis *strains W83 and ATCC33277 [28]. Also in this study we found these regions back in the analysis as described above

### Genes only aberrant in FDC381

FDC381 is the only strain included in this study that does not produce CPS. It is also the least virulent strain in mouse studies. Here, an analysis was performed to find genes that are specifically aberrant in FDC381 and not in all the other test strains (Table 7). Alongside many genes encoding hypothetical proteins several genes of special interest were found. The genes *PG1711 *encoding an alpha-1,2-mannosidase family protein, and *PG1972 *encoding the hemagglutinin *hagB*, all thought to be involved in virulence either by a role in evasion of the immune system or by a role in adhesion to host cells [29,59].

**Table 7:** Genes only aberrant in strain FDC381

GeneID	Annotated function
*PG0183*	lipoprotein, putative
*PG0204*	hypothetical protein
*PG0300*	TPR domain protein
*PG0492*	hypothetical protein
*PG1119*	flavodoxin, putative
*PG1199*	hypothetical protein
*PG1200*	hypothetical protein
*PG1373*	hypothetical protein
*PG1466*	hypothetical protein
*PG1467*	methlytransferase, UbiE-COQ5 family
*PG1473*	conjugative transposon protein TraQ
*PG1685*	hypothetical protein
*PG1711*	alpha-1,2-mannosidase family protein
*PG1777*	conserved hypothetical protein
*PG1786*	hypothetical protein
*PG1814*	DNA primase
*PG1969*	hypothetical protein
*PG1970*	hypothetical protein
*PG1972*	hemagglutinin protein HagB
*PG1977*	hypothetical protein
*PG1978*	hypothetical protein

Although these data do not directly show any CPS biosynthesis specific genes aberrant only in the non-encapsulated FDC381 it does give hints towards other virulence associated traits that are missing in FDC381.

### High versus lower virulence strains

When comparing the core gene set of only the highly virulent strains W83, HG1025, ATCC49417 and HG1690 with the genes aberrant in each of the less virulent strains HG184, HG1691, 34-4 and FDC381 an interesting result was seen. There is only a single gene, *hmuS, *that is present in all highly virulent strains but aberrant in each of the less virulent strains. *HmuS *is part of the *hmuYRSTUV *haemin uptake system [60]. Haemin acquisition is vital for *P. gingivalis *which makes the haemin uptake and storage system relevant study objects. Lacking part of an important uptake mechanism could have consequences for infection and survival. However, in these experiments no functional differences have been shown.

## Conclusions

In this study we analyzed the genetic contents of representative strains of each of the seven capsular serotypes. Comparative genomic hybridization shows that gene aberrance among *P. gingivalis *strains can be up to 13.7%, which is higher than previously reported. The *P. gingivalis *genome is variable with 20% of the W83 gene content being aberrant in at least one of the seven test strains. Analysis of virulence-related genes conservation was performed; only a few virulence-related genes were shown to be aberrant among test strains. As could be expected due to the choice of strains it was found that among the most aberrant virulence genes were the CPS biosynthesis genes.

In this study we initiated the description of a core genome of the anaerobic bacterium *P. gingivalis*, one of the most important causative agents of periodontitis allowing a more focused search for potential important virulence factors of which several were identified

## Methods

### Bacterial strains and maintenance

*P. gingivalis *strains used in this study are listed in Table 1, including serotype, origin and virulence level. *P. gingivalis *strains were first grown on 5% horse blood agar plates (Oxoid no. 2, Basingstoke, UK) supplemented with hemin (5 μg/ml) and menadione (1 μg/ml) (BA+H/M plates) at 37°C in an anaerobic atmosphere of 80% N_2_, 10% H_2_, and 10% CO_2_. From these plates 10 ml of liquid brain heart infusion broth supplemented with hemin (5 μg/ml) and menadione (1 μg/ml) (BHI+H/M) was inoculated and grown overnight as a pre-culture at 37°C in an anaerobic atmosphere. From the pre-culture a 300 ml 1:100 dilution in BHI+H/M was made, which was grown overnight at 37°C in an anaerobic atmosphere. The bacteria were washed 3 times in phosphate-buffered saline (PBS) and then pelleted and stored at -80°C until DNA isolation was performed.

### Microarray design

Whole-genome microarrays made for *P. gingivalis *strain W83 kindly provided by the Pathogen Functional Genomics Resource Center (The Institute for Genomic Research (TIGR), Rockville, MD) were used in this study. The aminosilane-coated microarrays contain 1,907 70-mer oligonucleotide probes designed on the 1,990 annotated W83 ORFs as found by TIGR. Each probe was designed to be unique for an ORF, so ORFs that were not unique were excluded. The arrays also included 500 *Arabidopsis thaliana *control probes. Each probe was printed four times on an array. Specific information about the microarrays can be found at http://pfgrc.jcvi.org/index.php/microarray/array_description/porphyromonas_gingivalis/version1.html

### DNA isolation

*P. gingivalis *pellets were frozen at -80°C until DNA isolation. Frozen pellets were ground in liquid nitrogen with a mortar and pestle until a fine powder was obtained. 500 μl of this powder was transferred to a liquid nitrogen pre-chilled 15 ml tube. DNA was extracted by addition of 1500 μl 65°C CTAB extraction buffer made to 2% (v/v) 2-mercaptoethanol before use (100 mM Tris-Cl (pH 8.0), 2.0 M NaCl, 20 mM EDTA, 3% (w/v) CTAB (H6269, Sigma-Aldrich), 2% (w/v) PVP-40 (PVP40, Sigma-Aldrich); Filter sterilized and stored at room temperature). After incubation for 30 min at 65°C with occasional mixing, DNA was extracted with 1500 μl phenol/chloroform/isoamylalcohol (25:24:1) (pH 7.9) (AM9730, Ambion). After centrifugation at 6,000 × g for 15 min, the aqueous phase was transferred to a clean 15 ml tube and DNA was precipitated with an equal volume of ice-cold isopropanol. DNA was pelleted at 6,000 × g for 15 min. The DNA pellet was washed twice with ice-cold 70% ethanol and centrifugation at 6,000 × g for 5 min. The remaining liquid was removed by decanting and the pellet was air dried. This pellet was resuspended in 600 μl TE and 1 μl RNAse A (10 mg/ml, R6513, Sigma-Aldrich) was added. Residual RNA was removed by overnight incubation at 37°C and DNA was re-extracted with an equal volume of phenol/chloroform/isoamylalcohol (25:24:1) pH 7.9. The aqueous phase was recovered by centrifugation at 6,000 × g for 15 min. The aqueous layer was treated with an equal volume of chloroform/IAA (96:4) and centrifuged at 6,000 × g for 10 min at room temperature. The final aqueous phase was treated with an equal volume of 100% ethanol and 1/10 volume of 3 M sodium acetate (pH 5.2) and incubated for 30 min @ -20°C. DNA was pelleted for 15 min at 6,000 × g. Residual liquid was removed and the pellet was washed once with ice-cold 70% ethanol. DNA was pelleted for 5 min at 6,000 × g and the pellet was air-dried. The DNA pellet was resuspended in an appropriate volume of TE. DNA quality was verified with gel electrophoresis (0.5% agarose in TAE).

### Genomic DNA labelling, microarray hybridization, scanning and data extraction

1 μg of genomic DNA was labeled with Cy3 or Cy5 using the CGH labeling kit for oligo arrays (ENZO Life Sciences). Labeled genomic DNA was purified with the QiaQuick PCR purification kit (Qiagen). *P. gingivalis *(W83) version 1 arrays were obtained from the Pathogen Functional Genomics Resource Center (PFGRC). Individual arrays were hybridized with 5 μg Cy3- and 5 μg Cy5-labeled material (test strains versus FDC381, which served as common reference), without dye swap, according to the Oligonucleotide Array-Based CGH for Genomic DNA Analysis manual (Agilent Technologies version 5.0). Briefly, labeled DNA was combined with 52 μl 10 × Blocking Agent and 260 μl 2 × Gex Hybridization Buffer Hi-RPM (Gene Expression Hybridization Kit, Agilent Technologies) in a total volume of 520 μl. Hybridization samples were incubated at 95°C for 3 min, spun down and hybridized at 37°C for 30 min. Samples were spun down and 490 μl of each sample was loaded onto a 1 × 244 k backing in a SureHyb hybridization chamber (Agilent Technologies) and a *P. gingivalis *version 1 array was placed on top. Hybridization was performed at 65°C for 24 h and 10 RPM in a hybridization oven (G2545A, Agilent Technologies). After the hybridization the backings were removed in LSW (2 × SSC, 0.1% Sarkosyl (L9150, Sigma-Aldrich) at room temperature, washed for 5 min at 42°C in LSW, washed for 10 min at room temperature in HSW (0.1 × SSC, 0.1% Sarkosyl) and finally washed for 1 min at room temperature in FW (0.1 × SSC). Each array was dipped 5 times in H_2_O and quickly submerged in isopropanol. Microarrays were spun dry for 1 min at 232 × g and scanned on an Agilent G2505B scanner at 5 μm resolution and data was extracted with Feature Extraction version 9.5.3.1. (Protocol GE2-NonAT_95_Feb07).

### Experimental design and Microarray data analysis

Each strain was cultured in triplicate, in three experimental batches. DNA isolations and hybridizations were therefore performed three times for each strain, each being a biological replicate analyzed in one experimental block. On each array four technical replicate spots were spotted.

After log2 transformation, the data was normalized by a global Lowess smoothing procedure, omitting the probes with highly divergent intensities because of the bias they induced. A mixed ANOVA model (as described in [61]) with group-means-parameterization was used to normalize the data and collapse the technical and biological replicates. The gene specific model was:

(1) $$y_{iklmn}=\mu +\tau_{i}+\rho +S_{k}+A_{l}+B_{m}+\varepsilon_{iklmn}$$

*y*_*ijklmn *_represents log2 expression intensities, *μ *is the gene specific mean, τ represents fixed strain effects (*i *= 1, ..., 8), *ρ *is an indicator variable indicating the common reference, *S *represents random spot effects (*j *= 1, ..., 96), *A *represents random array effects (*i *= 1, ..., 24), and *B *represents experimental batch effects (*m *= 1, ..., 3). Normalized average (Cy5) intensities for each strain were calculated as *y*_i_* = *μ *+ *τ*_*i *_and normalized average log2-ratio's with respect to W83 were calculated as *Y*_i_* = *τ*_*i *_- *τ*_*1*_, for each *i *≠ 1 (which represents W83).

Hence, each strain was compared with W83, and deviations in log2-ratio's were interpreted as aberrations. Given *j *genes divergence from zero were modelled as posterior probabilities of change under a mixture model, where non-divergent *Y*_ij_* ~ *N*(0,*s*_*i*_^2^) and divergent *Y*_ij_* follows a uniform distribution [62].

Highly variable regions due to mutations or loss were quantified according to [63], using their GLAD (Gain and Loss Analysis of DNA) package with default parameter settings. Finally, we used the negative control probes from *Arabidopsis thaliana *to define absent calls with the aim to quantify whether an aberration was found more likely due to mutation or loss. The distributions of intensities suggested a distinguishable mixed distribution of intensities from probes interrogating present genes (high) and probes interrogating absent genes (low; Figure 1). Given *j *probes, probe intensities were modelled using a standard Gaussian mixture model where probes interrogating present genes *y*_ij_* ~ *N*_1_(*μ*_1__*i*_,*s*_1__*i*_^2^) and probes interrogating absent genes *y*_ij_* ~ *N*_2_(*μ*_2__*i*_,*s*_2__*i*_^2^). The probe specific membership probabilities of *N*_1_(*μ*_1__*i*_,*s*_1__*i*_^2^) represents the null-hypothesis of "not absent", which is the hypothesis under test. False discovery rate correction as described by [64] was applied to both the test for quantifying aberrations as well as to the test for quantifying genomic losses. The data was visualized using the Integrated Genome Browser [65]. The final data set including dead probes and conserved, aberrant and absent genes is shown in additional file 3.

## Abbreviations

AFLP: amplified fragment length polymorphism; BA+H/M: Horse blood agar plates supplemented with hemin and menadione; CDSs: Coding sequences; CGH: Comparative genomic hybridization; CPS: Capsular polysaccharide; LPS: Lipopolysaccharide; OMP: Outer membrane protein; ORF: Open reading frame; PBS: Phosphate-buffered saline; SSC: Saline-sodium citrate buffer; TAE: Tris-acetate-EDTA buffer; TE: Tris-EDTA buffer.

## Authors' contributions

JB performed the microbiology and wrote the microbiological part of the manuscript. MdJ performed the DNA isolations and hybridizations. MJJ developed and performed the analysis methods and wrote part of the manuscript. FRAW was involved in study design and writing the manuscript. TMB, MLL, HdS were all involved in the design of the study. WC was involved in study design, supervision and drafting the paper. All authors read and approved the final manuscript.

## Supplementary Material

Additional file 1 **Conserved core gene set of *P. gingivalis***. The conserved core genes of *P. gingivalis *consisting of 1476 genes and two ambiguous genes, which are called non-aberrant but absent. Click here for file

Additional file 2 **W83-specific genes 65 genes**. aberrant in each test strain of which 39 W83-specific genes (marked in red) Click here for file

Additional file 3 ***P. gingivalis *CGH data set. **Table listing each *P. gingivalis *probe included in the results of this study in the order of geneID, including annotation. Low adjP-values (<0.05) depicted in yellow indicate aberrance in a test strain. High adj Pvals. absent (>0.99) depicted in red indicate absence in the test strain. Black rows indicate the dead probes as found on the W83 array in this study. Zooming out gives an overview of the whole genomic diversity along the test strains. Click here for file
