# Supplementary material for: The core genome of the anaerobic oral pathogenic bacterium Porphyromonas gingivalis
Source: BMC Microbiol. 2010 Sep 29;10:252. doi: 10.1186/1471-2180-10-252 (PMC2955634; doi:10.1186/1471-2180-10-252)
Supplement: Additional file 1 — Conserved core gene set of P. gingivalis. The conserved core genes of P. gingivalis consisting of 1476 genes and two ambiguous genes, which are called non-aberrant but absent. [file 1471-2180-10-252-S1.DOC]

### Additional file 1

Title**:** Conserved core gene set of *P. gingivalis* consisting of 1476 genes and two ambiguous genes

| **GeneID** | **description** |
| --- | --- |
| *PG0001* | chromosomal replication initiator protein DnaA |
| *PG0002* | hexapeptide transferase family protein |
| *PG0003* | membrane protein, putative |
| *PG0004* | transcriptional regulator, Sir2 family |
| *PG0005* | conserved hypothetical protein |
| *PG0006* | MATE efflux family protein |
| *PG0007* | hypothetical protein |
| *PG0010* | ATP-dependent Clp protease, ATP-binding subunit ClpC |
| *PG0011* | glycosyl hydrolase, family 3 |
| *PG0012* | L-threonine-O-3-phosphate decarboxylase, putative |
| *PG0013* | conserved hypothetical protein |
| *PG0014* | ISPg2, transposase, truncation |
| *PG0016* | sigma-54 dependent DNA-binding response regulator |
| *PG0017* | sensor histidine kinase |
| *PG0018* | hypothetical protein |
| *PG0020* | transcriptional regulator, MarR family |
| *PG0021* | TIM-barrel protein, putative, NifR3 family |
| *PG0022* | sulfate permease family protein |
| *PG0024* | DNA-binding protein, putative |
| *PG0025* | fumarylacetoacetate hydrolase family protein |
| *PG0026* | hypothetical protein |
| *PG0027* | hypothetical protein |
| *PG0028* | 2C-methyl-D-erythritol 2,4-cyclodiphosphate synthase |
| *PG0029* | transposase, truncation-RNA methyltransferase, TrmH family, truncation |
| *PG0030* | cytidine deaminase |
| *PG0032* | beta-mannosidase, putative |
| *PG0033* | RmuC domain protein |
| *PG0034* | thioredoxin |
| *PG0035* | DNA polymerase III, alpha subunit |
| *PG0037* | ribosomal protein L19 |
| *PG0039* | hypothetical protein |
| *PG0042* | serine hydroxymethyltransferase |
| *PG0043* | beta-hexosaminidase |
| *PG0045* | heat shock protein HtpG |
| *PG0046* | phosphatidate cytidylyltransferase |
| *PG0047* | cell division protein FtsH, putative |
| *PG0048* | conserved hypothetical protein TIGR00092 |
| *PG0049* | hypothetical protein |
| *PG0052* | sensor histidine kinase |
| *PG0053* | hypothetical protein |
| *PG0054* | single-stranded-DNA-specific exonuclease RecJ |
| *PG0055* | conserved domain protein |
| *PG0056* | hypothetical protein |
| *PG0057* | nicotinate phosphoribosyltransferase |
| *PG0058* | nicotinate (nicotinamide) nucleotide adenylyltransferase |
| *PG0059* | hypothetical protein |
| *PG0060* | hypothetical protein |
| *PG0061* | yngK protein |
| *PG0062* | TPR domain protein |
| *PG0063* | outer membrane efflux protein |
| *PG0064* | heavy metal efflux pump, CzcA family |
| *PG0065* | efflux transporter, RND family, MFP subunit |
| *PG0066* | hypothetical protein |
| *PG0068* | hypothetical protein |
| *PG0069* | conserved hypothetical protein |
| *PG0070* | acyl-(acyl-carrier-protein)-UDP-N-acetylglucosamine acyltransferase |
| *PG0071* | UDP-3-O-acyl-GlcNAc deacetylase-beta-hydroxyacyl-[acyl carrier protein] dehydratase FabZ |
| *PG0072* | UDP-3-O-[3-hydroxymyristoyl] glucosamine N-acyltransferase |
| *PG0073* | orotidine 5-monophosphate decarboxylase |
| *PG0074* | peptide chain release factor 1 |
| *PG0075* | phosphoribosylformylglycinamidine cyclo-ligase, putative |
| *PG0076* | N-acetylmuramoyl-L-alanine amidase, family 4 |
| *PG0080* | hypothetical protein |
| *PG0081* | hypothetical protein |
| *PG0082* | hypothetical protein |
| *PG0083* | hypothetical protein |
| *PG0084* | L-serine dehydratase, iron-sulfur-dependent, single chain form |
| *PG0085* | alpha-galactosidase |
| *PG0086* | ATP-dependent RNA helicase, DEAD-DEAH box family |
| *PG0087* | SIS domain protein |
| *PG0088* | peptidase, M16 family |
| *PG0090* | Dps family protein |
| *PG0091* | transporter, putative |
| *PG0092* | transporter, putative |
| *PG0094* | outer membrane efflux protein, putative |
| *PG0095* | DNA mismatch repair protein MutS |
| *PG0099* | phenylalanyl-tRNA synthetase, beta subunit |
| *PG0104* | DNA topoisomerase III |
| *PG0106* | glycosyl transferase, group 4 family protein |
| *PG0108* | UDP-N-acetyl-D-mannosaminuronic acid dehydrogenase |
| *PG0121* | DNA-binding protein HU |
| *PG0123* | hypothetical protein |
| *PG0124* | conserved hypothetical protein |
| *PG0126* | type I phosphodiesterase-nucleotide pyrophosphatase family protein |
| *PG0127* | ferrochelatase |
| *PG0129* | mannosyltransferase |
| *PG0130* | phosphoglycerate mutase |
| *PG0132* | hypothetical protein |
| *PG0133* | hypothetical protein |
| *PG0134* | magnesium transporter |
| *PG0135* | dimethyladenosine transferase |
| *PG0136* | hypothetical protein |
| *PG0137* | aminoacyl-histidine dipeptidase |
| *PG0138* | malonyl CoA-acyl carrier protein transacylase |
| *PG0139* | membrane-bound lytic murein transglycosylase D, putative |
| *PG0140* | hypothetical protein |
| *PG0141* | spoOJ protein |
| *PG0142* | SpoOJ regulator protein |
| *PG0143* | hydrolase, carbon-nitrogen family |
| *PG0144* | conserved hypothetical protein |
| *PG0146* | hypothetical protein |
| *PG0147* | hypothetical protein |
| *PG0148* | sigma-54-dependent transcriptional regulator |
| *PG0149* | conserved domain protein |
| *PG0150* | conserved hypothetical protein TIGR01125 |
| *PG0151* | signal recognition particle-docking protein FtsY |
| *PG0152* | carboxynorspermidine decarboxylase |
| *PG0153* | aspartyl-tRNA synthetase |
| *PG0155* | riboflavin biosynthesis protein RibD |
| *PG0156* | modification methylase, HemK family |
| *PG0157* | regulatory protein RecX |
| *PG0158* | competence protein F-related protein |
| *PG0159* | endopeptidase PepO |
| *PG0160* | conserved domain protein |
| *PG0161* | hypothetical protein |
| *PG0162* | RNA polymerase sigma-70 factor, ECF subfamily |
| *PG0163* | phosphofructokinase |
| *PG0164* | conserved hypothetical protein |
| *PG0165* | heat shock protein 15 |
| *PG0166* | peptidyl-tRNA hydrolase |
| *PG0167* | ribosomal protein L25 |
| *PG0170* | methionyl-tRNA synthetase |
| *PG0171* | 5-nucleotidase family protein |
| *PG0172* | exonuclease |
| *PG0173* | transcriptional regulator, putative |
| *PG0188* | lipoprotein, putative |
| *PG0189* | hypothetical protein |
| *PG0190* | undecaprenyl diphosphate synthase |
| *PG0191* | outer membrane protein, putative |
| *PG0192* | cationic outer membrane protein OmpH |
| *PG0193* | cationic outer membrane protein OmpH |
| *PG0195* | rubrerythrin |
| *PG0196* | peptidase, M16 family |
| *PG0199* | TatD family protein |
| *PG0200* | conserved hypothetical protein TIGR00278 |
| *PG0201* | ribonuclease P protein component |
| *PG0202* | uroporphyrinogen-III synthase HemD, putative |
| *PG0203* | hypothetical protein |
| *PG0205* | peptide chain release factor 3 |
| *PG0209* | formate-nitrite transporter |
| *PG0210* | precorrin-6x reductase-cobalamin biosynthetic protein CbiD |
| *PG0211* | cobalamin biosynthesis protein CbiG-precorrin-4 C11-methyltransferase |
| *PG0213* | precorrin-3 methylase-precorrin-8X methylmutase |
| *PG0214* | RNA polymerase sigma-70 factor, ECF subfamily |
| *PG0215* | hypothetical protein |
| *PG0216* | hypothetical protein |
| *PG0217* | hypothetical protein |
| *PG0218* | hypothetical protein |
| *PG0223* | exonuclease |
| *PG0224* | conserved hypothetical protein |
| *PG0226* | transglutaminase-related protein |
| *PG0227* | DNA repair protein RadA |
| *PG0228* | DdaH family protein |
| *PG0229* | hypothetical protein |
| *PG0230* | transaldolase TalC, putative |
| *PG0231* | conserved hypothetical protein |
| *PG0232* | zinc carboxypeptidase, putative |
| *PG0234* | immunoreactive 23 kDa antigen PG66 |
| *PG0235* | carboxyl-terminal protease |
| *PG0236* | hypothetical protein |
| *PG0237* | uracil-DNA glycosylase |
| *PG0240* | hydrolase, haloacid dehalogenase-like family |
| *PG0241* | lipoprotein, putative |
| *PG0242* | conserved hypothetical protein TIGR00096 |
| *PG0243* | hypothetical protein |
| *PG0245* | universal stress protein family |
| *PG0246* | hypothetical protein |
| *PG0248* | translation initation factor SUI1, putative |
| *PG0249* | oxaloacetate decarboxylase, putative |
| *PG0253* | conserved hypothetical protein |
| *PG0254* | N utilization substance protein A, putative |
| *PG0255* | translation initiation factor IF-2 |
| *PG0256* | CvpA family protein |
| *PG0257* | conserved hypothetical protein |
| *PG0258* | ABC transporter, ATP-binding protein |
| *PG0259* | conserved hypothetical protein |
| *PG0263* | tyrosyl-tRNA synthetase |
| *PG0264* | glycosyl transferase, group 2 family protein |
| *PG0265* | hypothetical protein |
| *PG0267* | arginyl-tRNA synthetase |
| *PG0268* | tRNA (5-methylaminomethyl-2-thiouridylate)-methyltransferase |
| *PG0269* | exodeoxyribonuclease III |
| *PG0270* | redox-sensitive transcriptional activator OxyR |
| *PG0271* | single-stranded binding protein |
| *PG0272* | CBS domain protein |
| *PG0273* | 4-phosphopantetheinyl transferase family protein |
| *PG0274* | hypothetical protein |
| *PG0275* | thioredoxin family protein |
| *PG0276* | conserved hypothetical protein |
| *PG0278* | hypothetical protein |
| *PG0279* | NADP-dependent malic enzyme |
| *PG0281* | ABC transporter, permease protein, putative |
| *PG0282* | ABC transporter, ATP-binding protein |
| *PG0283* | efflux transporter, MFP component, RND family |
| *PG0285* | hypothetical protein |
| *PG0286* | hypothetical protein |
| *PG0287* | hypothetical protein |
| *PG0288* | lipoprotein, putative |
| *PG0289* | hypothetical protein |
| *PG0290* | hypothetical protein |
| *PG0291* | hypothetical protein |
| *PG0293* | secretion activator protein, putative |
| *PG0294* | glycosyl transferase, group 2 family protein |
| *PG0295* | DNA processing protein DprA, putative |
| *PG0296* | phosphoribosylformylglycinamidine synthase |
| *PG0302* | hypothetical protein |
| *PG0303* | electron transport complex, RnfABCDGE type, B subunit |
| *PG0304* | electron transport complex, RnfABCDGE type, C subunit |
| *PG0305* | electron transport complex, RnfABCDGE type, D subunit |
| *PG0306* | electron transport complex, RnfABCDGE type, G subunit |
| *PG0307* | electron transport complex, RnfABCDGE type, E subunit |
| *PG0308* | electron transport complex, RnfABCDGE type, A subunit |
| *PG0309* | thiamine biosynthesis lipoprotein ApbE |
| *PG0310* | nitroreductase family protein |
| *PG0311* | glycosyl transferase, group 2 family protein |
| *PG0312* | hypothetical protein |
| *PG0313* | hypothetical protein |
| *PG0314* | ribosomal protein L21 |
| *PG0315* | ribosomal protein L27 |
| *PG0316* | seryl-tRNA synthetase |
| *PG0319* | hypothetical protein |
| *PG0320* | hypothetical protein |
| *PG0321* | LAO-AO transport system ATPase |
| *PG0322* | serine-threonine transporter |
| *PG0323* | conserved hypothetical protein |
| *PG0324* | histidine ammonia-lyase |
| *PG0325* | conserved hypothetical protein |
| *PG0326* | hypothetical protein |
| *PG0327* | hypothetical protein |
| *PG0328* | imidazolonepropionase |
| *PG0329* | formiminotransferase-cyclodeaminase-related protein |
| *PG0330* | DNA-binding protein, histone-like family |
| *PG0332* | transcription termination factor Rho |
| *PG0333* | membrane protein, putative |
| *PG0334* | glycosyl transferase, group 2 family protein |
| *PG0335* | tRNA delta(2)-isopentenylpyrophosphate transferase |
| *PG0336* | hypothetical protein |
| *PG0337* | hypothetical protein |
| *PG0338* | hypothetical protein |
| *PG0339* | hypothetical protein |
| *PG0343* | methionine gamma-lyase |
| *PG0344* | purple acid phosphatase |
| *PG0345* | hypothetical protein |
| *PG0346* | GTP-binding protein |
| *PG0347* | UDP-glucose 4-epimerase |
| *PG0348* | ATP-dependent DNA helicase RecG |
| *PG0350* | internalin-related protein |
| *PG0351* | hypothetical protein |
| *PG0352* | sialidase, putative |
| *PG0355* | hypothetical protein |
| *PG0356* | conserved hypothetical protein |
| *PG0357* | aspartate carbamoyltransferase, catalytic subunit |
| *PG0358* | aspartate carbamoyltransferase, regulatory subunit |
| *PG0359* | flavin reductase domain protein |
| *PG0360* | lemA protein |
| *PG0361* | conserved domain protein |
| *PG0362* | hypothetical protein |
| *PG0363* | conserved domain protein |
| *PG0364* | conserved hypothetical protein |
| *PG0365* | 3-5 exonuclease domain protein |
| *PG0366* | hypothetical protein |
| *PG0368* | DNA topoisomerase IV, B subunit, putative |
| *PG0369* | phosphopantetheine adenylyltransferase |
| *PG0371* | hypothetical protein |
| *PG0373* | hypothetical protein |
| *PG0374* | hypothetical protein |
| *PG0376* | ribosomal protein S9 |
| *PG0377* | ribosomal protein S2 |
| *PG0378* | translation elongation factor Ts |
| *PG0380* | excinuclease ABC, B subunit |
| *PG0381* | sodium-hydrogen antiporter |
| *PG0383* | membrane-associated zinc metalloprotease, putative |
| *PG0384* | MutS2 family protein |
| *PG0385* | ribosomal protein S21 |
| *PG0386* | site-specific recombinase, phage integrase family-ribosomal subunit interface protein |
| *PG0387* | translation elongation factor Tu |
| *PG0389* | transcription antitermination protein NusG |
| *PG0390* | ribosomal protein L11 |
| *PG0391* | ribosomal protein L1 |
| *PG0392* | ribosomal protein L10 |
| *PG0393* | ribosomal protein L7-L12 |
| *PG0394* | DNA-directed RNA polymerase, beta subunit |
| *PG0395* | DNA-directed RNA polymerase, beta subunit |
| *PG0396* | transcriptional regulator, Crp-Fnr family |
| *PG0397* | hypothetical protein |
| *PG0398* | recF protein |
| *PG0399* | lipoprotein, putative |
| *PG0400* | conserved hypothetical protein |
| *PG0401* | KH-HDIG domain protein |
| *PG0403* | hypothetical protein |
| *PG0404* | hypothetical protein |
| *PG0408* | hypothetical protein |
| *PG0409* | hypothetical protein |
| *PG0412* | DNA mismatch repair protein MutL |
| *PG0413* | hypothetical protein |
| *PG0414* | hypothetical protein |
| *PG0415* | peptidyl-prolyl cis-trans isomerase, PPIC-type |
| *PG0416* | ATP-dependent DNA helicase RecQ |
| *PG0417* | ATP-dependent Clp protease, ATP-binding subunit ClpX |
| *PG0418* | ATP-dependent Clp protease, proteolytic subunit |
| *PG0419* | hypothetical protein |
| *PG0422* | hypothetical protein |
| *PG0423* | hypothetical protein |
| *PG0424* | hypothetical protein |
| *PG0425* | esterase, putative |
| *PG0428* | hypothetical protein |
| *PG0429* | pyruvate synthase |
| *PG0430* | oxidoreductase, putative |
| *PG0431* | hypothetical protein |
| *PG0432* | NOL1-NOP2-sun family protein |
| *PG0433* | tetrapyrrole methylase family protein |
| *PG0434* | hypothetical protein |
| *PG0435* | capsular polysaccharide biosythesis protein, putative |
| *PG0437* | polysaccharide export protein, BexD-CtrA-VexA family |
| *PG0438* | hypothetical protein |
| *PG0441* | hypothetical protein |
| *PG0443* | hemagglutinin-related protein |
| *PG0445* | peptidase T |
| *PG0446* | thiF protein |
| *PG0447* | conserved hypothetical protein |
| *PG0448* | hypothetical protein |
| *PG0449* | TPR domain protein |
| *PG0450* | hypothetical protein |
| *PG0451* | CBS domain protein |
| *PG0452* | conserved hypothetical protein |
| *PG0453* | conserved domain protein |
| *PG0462* | transporter, putative |
| *PG0463* | folylpolyglutamate synthase |
| *PG0464* | adenylosuccinate synthetase |
| *PG0465* | ferric uptake transcriptional regulator |
| *PG0466* | hypothetical protein |
| *PG0468* | mannose-6-phosphate isomerase, class I |
| *PG0469* | hypothetical protein |
| *PG0470* | hypothetical protein |
| *PG0471* | hypothetical protein |
| *PG0472* | iron-sulfur cluster binding protein, putative |
| *PG0474* | low-specificity L-threonine aldolase |
| *PG0475* | oxygen-independent coproporphyrinogen III oxidase, putative |
| *PG0476* | yngK protein |
| *PG0477* | pantoate--beta-alanine ligase |
| *PG0479* | hypothetical protein |
| *PG0481* | 2-amino-3-ketobutyrate CoA ligase |
| *PG0482* | hypothetical protein |
| *PG0483* | kinase, putative |
| *PG0484* | hypothetical protein |
| *PG0485* | preprotein translocase, YajC subunit |
| *PG0486* | methylated-DNA--protein-cysteine S-methyltransferase |
| *PG0488* | Holliday junction DNA helicase RuvB |
| *PG0489* | polysaccharide biosynthesis-related protein |
| *PG0490* | membrane protein, putative |
| *PG0491* | conserved hypothetical protein |
| *PG0494* | hypothetical protein |
| *PG0495* | hypothetical protein |
| *PG0496* | hypothetical protein |
| *PG0497* | 5-methylthioadenosine-S-adenosylhomocysteine nucleosidase |
| *PG0500* | queuine tRNA-ribosyltransferase |
| *PG0501* | conserved hypothetical protein |
| *PG0502* | SsrA-binding protein |
| *PG0503* | dipeptidyl aminopeptidase IV |
| *PG0504* | lipoate synthase |
| *PG0505* | hypothetical protein |
| *PG0506* | arginine-specific cysteine proteinase |
| *PG0508* | HAD-superfamily subfamily IB hydrolase, TIGR01490 |
| *PG0509* | prenyltransferase, UbiA family |
| *PG0510* | conserved hypothetical protein |
| *PG0511* | spore maturation protein A-spore maturation protein B |
| *PG0512* | guanylate kinase |
| *PG0513* | conserved hypothetical protein TIGR00255 |
| *PG0514* | preprotein translocase, SecA subunit |
| *PG0515* | conserved hypothetical protein |
| *PG0516* | conserved hypothetical protein |
| *PG0517* | hypothetical protein |
| *PG0518* | abortive infection protein family |
| *PG0519* | hypothetical protein |
| *PG0520* | chaperonin, 60 kDa |
| *PG0521* | chaperonin, 10 kDa |
| *PG0522* | tRNA delta(2)-isopentenylpyrophosphate transferase |
| *PG0523* | inosine-5-monophosphate dehydrogenase |
| *PG0524* | hypothetical protein |
| *PG0525* | CTP synthase |
| *PG0526* | membrane protein, putative |
| *PG0528* | amidophosphoribosyltransferase, putative |
| *PG0529* | carbamoyl-phosphate synthase, small subunit |
| *PG0530* | carbamoyl-phosphate synthase, large subunit |
| *PG0531* | glutamine-dependent NAD+ synthetase |
| *PG0532* | conserved domain protein |
| *PG0534* | hypothetical protein |
| *PG0535* | conserved hypothetical protein |
| *PG0536* | hypothetical protein |
| *PG0537* | aminoacyl-histidine dipeptidase |
| *PG0538* | outer membrane efflux protein |
| *PG0539* | efflux transporter, MFP component, RND family |
| *PG0540* | AcrB-AcrD-AcrF family protein |
| *PG0541* | hypothetical protein |
| *PG0547* | conserved hypothetical protein |
| *PG0548* | pyruvate ferredoxin-flavodoxin oxidoreductase family protein |
| *PG0553* | extracellular protease, putative |
| *PG0554* | hypothetical protein |
| *PG0555* | DNA-binding protein, histone-like family |
| *PG0558* | purine nucleoside phosphorylase I, inosine and guanosine-specific |
| *PG0559* | chlorohydrolase family protein |
| *PG0561* | peptidase, M20-M25-M40 family |
| *PG0562* | potassium uptake protein TrkA, putative |
| *PG0568* | translation elongation factor P |
| *PG0571* | aspartate-semialdehyde dehydrogenase |
| *PG0572* | hypothetical protein |
| *PG0573* | S-adenosyl-methyltransferase MraW |
| *PG0574* | hypothetical protein |
| *PG0575* | penicillin-binding protein 2, putative |
| *PG0576* | UDP-N-acetylmuramoylalanyl-D-glutamyl-2, 6-diaminopimelate ligase |
| *PG0577* | phospho-N-acetylmuramoyl-pentapeptide-transferase |
| *PG0578* | UDP-N-acetylmuramoylalanine--D-glutamate ligase |
| *PG0579* | cell division protein FtsW, putative |
| *PG0580* | UDP-N-acetylglucosamine--N-acetylmuramyl-(pentapeptide) pyrophosphoryl-undecaprenol N-acetylglucosamine transferase |
| *PG0581* | UDP-N-acetylmuramate--alanine ligase |
| *PG0582* | cell division protein FtsQ, putative |
| *PG0583* | cell division protein FtsA |
| *PG0584* | cell division protein FtsZ |
| *PG0585* | YqeY family protein |
| *PG0587* | yadS protein |
| *PG0588* | 3-methyl-2-oxobutanoate hydroxymethyltransferase |
| *PG0589* | GMP synthase |
| *PG0592* | ribosomal protein L31 |
| *PG0593* | htrA protein |
| *PG0594* | RNA polymerase sigma-70 factor |
| *PG0595* | ribosomal protein S6 |
| *PG0596* | ribosomal protein S18 |
| *PG0597* | ribosomal protein L9 |
| *PG0598* | hypothetical protein |
| *PG0599* | 3,4-dihydroxy-2-butanone 4-phosphate synthase-GTP cyclohydrolase II |
| *PG0602* | hypothetical protein |
| *PG0605* | hypothetical protein |
| *PG0606* | hypothetical protein |
| *PG0607* | hypothetical protein |
| *PG0608* | hypothetical protein |
| *PG0610* | hypothetical protein |
| *PG0612* | hypothetical protein |
| *PG0613* | hypothetical protein |
| *PG0615* | GTP-binding protein TypA |
| *PG0616* | thioredoxin, putative |
| *PG0618* | alkyl hydroperoxide reductase, C subunit |
| *PG0619* | alkyl hydroperoxide reductase, F subunit |
| *PG0620* | ATP-dependent protease La |
| *PG0621* | conserved hypothetical protein |
| *PG0622* | hypothetical protein |
| *PG0623* | triosephosphate isomerase |
| *PG0624* | hypothetical protein |
| *PG0625* | GTP cyclohydrolase I |
| *PG0627* | RNA-binding protein |
| *PG0628* | ABC transporter, ATP-binding protein |
| *PG0629* | ATP-NAD kinase |
| *PG0630* | pyridoxal phosphate biosynthetic protein PdxJ |
| *PG0631* | MotA-TolQ-ExbB proton channel family protein |
| *PG0632* | biopolymer transport protein ExbD, putative |
| *PG0633* | hypothetical protein |
| *PG0634* | ThiJ-PfpI family protein |
| *PG0635* | ribosomal protein L11 methyltransferase |
| *PG0636* | MATE efflux family protein |
| *PG0637* | thiamine monophosphate kinase |
| *PG0638* | tetraacyldisaccharide 4-kinase |
| *PG0639* | signal peptide peptidase SppA, 67K type |
| *PG0644* | TonB-linked receptor Tlr, authentic frameshift |
| *PG0645* | conserved domain protein |
| *PG0646* | iron compound ABC transporter, ATP-binding protein |
| *PG0647* | iron compound ABC transporter, permease protein |
| *PG0648* | iron compound ABC transporter, periplasmic iron compound-binding protein, putative |
| *PG0649* | hypothetical protein |
| *PG0650* | hypothetical protein |
| *PG0651* | HDIG domain protein |
| *PG0652* | conserved hypothetical protein |
| *PG0653* | phosphoserine phosphatase |
| *PG0654* | hypothetical protein |
| *PG0656* | ribosomal protein L34 |
| *PG0657* | maf protein |
| *PG0658* | phosphatase, YrbI family |
| *PG0659* | conserved hypothetical protein |
| *PG0660* | nitroreductase family protein |
| *PG0661* | hypothetical protein |
| *PG0664* | oxidoreductase, Gfo-Idh-MocA family |
| *PG0665* | beta-galactosidase |
| *PG0666* | mdsC protein, authentic frameshift |
| *PG0668* | TonB-dependent receptor |
| *PG0669* | heme-binding protein FetB |
| *PG0670* | lipoprotein, putative |
| *PG0671* | iron compound ABC transporter, permease protein |
| *PG0672* | iron compound ABC transporter, ATP-binding protein |
| *PG0674* | indolepyruvate ferredoxin oxidoreductase, beta subunit |
| *PG0675* | indolepyruvate ferredoxin oxidoreductase, alpha subunit |
| *PG0676* | oxidoreductase, short chain dehydrogenase-reductase family |
| *PG0677* | saccharopine dehydrogenase |
| *PG0678* | pyrazinamidase-nicotinamidase, putative |
| *PG0679* | outer membrane efflux protein |
| *PG0680* | efflux transporter, MFP component, RND family |
| *PG0682* | ABC transporter, permease protein, putative |
| *PG0684* | ABC transporter, permease protein, putative |
| *PG0685* | ABC transporter, ATP-binding protein |
| *PG0686* | conserved hypothetical protein |
| *PG0687* | succinate-semialdehyde dehydrogenase |
| *PG0689* | NAD-dependent 4-hydroxybutyrate dehydrogenase |
| *PG0690* | 4-hydroxybutyrate CoA-transferase |
| *PG0691* | NifU-related protein |
| *PG0692* | 4-hydroxybutyryl-CoA dehydratase |
| *PG0694* | immunoreactive 42 kDa antigen PG33 |
| *PG0695* | immunoreactive 43 kDa antigen PG32 |
| *PG0698* | lipoprotein, putative |
| *PG0700* | hypothetical protein |
| *PG0701* | cobinamide kinase-cobinamide phosphate guanylyltransferase |
| *PG0702* | nicotinate-nucleotide--dimethylbenzimidazole phosphoribosyltransferase, putative |
| *PG0703* | cobalamin (5-phosphate) synthase, putative |
| *PG0704* | phosphoglycerate mutase family protein |
| *PG0705* | glutamate racemase |
| *PG0706* | hypothetical protein |
| *PG0707* | TonB-dependent receptor, putative |
| *PG0708* | peptidyl-prolyl cis-trans isomerase, FKBP-type |
| *PG0709* | peptidyl-prolyl cis-trans isomerase FkpA, FKBP-type |
| *PG0710* | peptidyl-prolyl cis-trans isomerase, FKBP-type |
| *PG0711* | hypothetical protein |
| *PG0712* | hypothetical protein |
| *PG0713* | anthranilate synthase component II |
| *PG0714* | copper homeostasis protein CutC |
| *PG0715* | transporter |
| *PG0720* | DNA-binding response regulator |
| *PG0721* | NLP-P60 family protein |
| *PG0722* | hypothetical protein |
| *PG0723* | hypothetical protein |
| *PG0724* | prolyl oligopeptidase family protein |
| *PG0725* | hydrolase, haloacid dehalogenase-like family |
| *PG0726* | lipoprotein, putative |
| *PG0727* | hypothetical protein |
| *PG0728* | conserved hypothetical protein |
| *PG0729* | D-alanine--D-alanine ligase |
| *PG0730* | ribosomal large subunit pseudouridine synthase D |
| *PG0731* | hypothetical protein |
| *PG0732* | hypothetical protein |
| *PG0733* | riboflavin synthase, alpha subunit |
| *PG0734* | nitroreductase family protein |
| *PG0735* | aminotransferase, class V |
| *PG0736* | ribonuclease HII |
| *PG0737* | hypothetical protein |
| *PG0738* | cytidine-deoxycytidylate deaminase family protein |
| *PG0739* | metallo-beta-lactamase family protein |
| *PG0740* | NLP-P60 family protein |
| *PG0744* | RNA methyltransferase, TrmH family |
| *PG0745* | lactoylglutathione lyase, putative |
| *PG0746* | sensor histidine kinase |
| *PG0747* | sigma-54 dependent DNA-binding response regulator |
| *PG0749* | hypothetical protein |
| *PG0750* | glycosyl transferase, group 2 family protein |
| *PG0751* | porT protein |
| *PG0752* | uracil phosphoribosyltransferase, putative |
| *PG0753* | protease |
| *PG0754* | DNA topoisomerase I |
| *PG0756* | conserved hypothetical protein |
| *PG0757* | hypothetical protein |
| *PG0758* | peptidyl-dipeptidase Dcp |
| *PG0759* | TPR domain protein |
| *PG0762* | trigger factor, putative |
| *PG0766* | polyribonucleotide nucleotidyltransferase |
| *PG0767* | 4-alpha-glucanotransferase |
| *PG0768* | conserved hypothetical protein |
| *PG0769* | fibronectin type III domain protein |
| *PG0770* | hypothetical protein |
| *PG0771* | hypothetical protein |
| *PG0774* | hypothetical protein |
| *PG0775* | acyl-CoA dehydrogenase family protein |
| *PG0776* | electron transfer flavoprotein, alpha subunit |
| *PG0777* | electron transfer flavoprotein, beta subunit |
| *PG0778* | conserved hypothetical protein |
| *PG0779* | hypothetical protein |
| *PG0780* | hypothetical protein |
| *PG0781* | hypothetical protein |
| *PG0782* | MotA-TolQ-ExbB proton channel family protein |
| *PG0783* | hydrolase, putative |
| *PG0784* | polyprenyl synthetase |
| *PG0787* | hypothetical protein |
| *PG0788* | hypothetical protein |
| *PG0789* | conserved hypothetical protein |
| *PG0790* | GTP-binding protein Obg |
| *PG0791* | adenylate kinase |
| *PG0792* | hypoxanthine phosphoribosyltransferase |
| *PG0793* | fructose-1,6-bisphosphatase |
| *PG0794* | penicillin-binding protein 1A, putative |
| *PG0795* | membrane protein, putative |
| *PG0796* | leucyl-tRNA synthetase |
| *PG0799* | hypothetical protein |
| *PG0800* | conserved hypothetical protein |
| *PG0801* | polyA polymerase family protein |
| *PG0802* | alpha keto acid dehydrogenase complex, E3 component, lipoamide dehydrogenase |
| *PG0803* | glucosamine-6-phosphate isomerase |
| *PG0804* | flavodoxin |
| *PG0805* | prolipoprotein diacylglyceryl transferase |
| *PG0806* | oxidoreductase, Gfo-Idh-MocA family |
| *PG0807* | NusB family protein |
| *PG0810* | hypothetical protein |
| *PG0811* | Holliday junction DNA helicase RuvA |
| *PG0812* | ISPg9, transposase, degenerate |
| *PG0813* | ISPg1, transposase, truncation |
| *PG0876* | thiophene and furan oxidation protein ThdF |
| *PG0877* | hypothetical protein |
| *PG0881* | recA protein |
| *PG0882* | hypothetical protein |
| *PG0883* | hypothetical protein |
| *PG0884* | hypothetical protein |
| *PG0885* | phospho-2-dehydro-3-deoxyheptonate aldolase-chorismate mutase |
| *PG0886* | hypothetical protein |
| *PG0888* | hypothetical protein |
| *PG0889* | peptidase, M24 family |
| *PG0890* | alkaline phosphatase, putative |
| *PG0893* | prismane protein |
| *PG0894* | DNA repair protein RadC |
| *PG0896* | beta-galactosidase |
| *PG0897* | alpha-amylase family protein |
| *PG0898* | conserved hypothetical protein |
| *PG0899* | cytochrome d ubiquinol oxidase, subunit II |
| *PG0900* | cytochrome d ubiquinol oxidase, subunit I |
| *PG0901* | conserved hypothetical protein |
| *PG0902* | alpha-1,2-mannosidase family protein |
| *PG0903* | conserved hypothetical protein |
| *PG0906* | lipoprotein, putative |
| *PG0908* | G-U mismatch-specific DNA glycosylase, putative |
| *PG0909* | conserved hypothetical protein |
| *PG0910* | FHA domain protein |
| *PG0912* | polysaccharide transport protein, putative |
| *PG0914* | hypothetical protein |
| *PG0915* | conserved hypothetical protein |
| *PG0917* | GtrA family protein |
| *PG0918* | hypothetical protein |
| *PG0919* | dihydroorotase |
| *PG0920* | glycosyl transferase, group 2 family protein |
| *PG0922* | membrane protein, putative |
| *PG0923* | ribosome-binding factor A |
| *PG0924* | 5-nucleotidase, lipoprotein e(P4) family |
| *PG0925* | thymidine kinase |
| *PG0926* | hypothetical protein |
| *PG0927* | conserved hypothetical protein TIGR00150 |
| *PG0928* | response regulator |
| *PG0930* | hypothetical protein |
| *PG0931* | DNA-binding protein, histone-like family, degenerate |
| *PG0932* | DNA polymerase III, delta prime subunit, putative |
| *PG0933* | translation elongation factor G, putative |
| *PG0934* | radical SAM domain protein |
| *PG0935* | 4-diphosphocytidyl-2C-methyl-D-erythritol kinase |
| *PG0936* | xanthine-uracil permease family protein |
| *PG0937* | hypothetical protein |
| *PG0938* | calcium-transporting ATPase |
| *PG0945* | ABC transporter, permease protein, putative |
| *PG0946* | ABC transporter, ATP-binding protein |
| *PG0948* | AMP nucleosidase, putative |
| *PG0949* | conserved hypothetical protein |
| *PG0950* | glycine cleavage system H protein |
| *PG0951* | phosphoribosylaminoimidazole carboxylase, PurE protein |
| *PG0952* | 1-hydroxy-2-methyl-2-(E)-butenyl 4-diphosphate synthase |
| *PG0953* | deoxyuridine 5-triphosphate nucleotidohydrolase |
| *PG0955* | hypothetical protein |
| *PG0956* | peptidase, M23-M37 family, putative |
| *PG0957* | riboflavin biosynthesis protein RibF |
| *PG0958* | YihY family protein |
| *PG0959* | ATP-binding protein, Mrp-Nbp35 family |
| *PG0960* | conserved hypothetical protein |
| *PG0961* | hypothetical protein |
| *PG0962* | prolyl-tRNA synthetase |
| *PG0963* | hypothetical protein |
| *PG0964* | CDP-diacylglycerol--serine O-phosphatidyltransferase, putative, authentic point mutation |
| *PG0965* | phosphatidylserine decarboxylase-related protein |
| *PG0969* | S-adenosylmethionine:tRNA ribosyltransferase-isomerase, putative |
| *PG0973* | alpha-1,2-mannosidase family protein |
| *PG0975* | PhoH family protein |
| *PG0976* | phosphoribosylaminoimidazole-succinocarboxamide synthase, putative |
| *PG0977* | ubiquinone-menaquinone biosynthesis methyltransferase UbiE |
| *PG0978* | shikimate 5-dehydrogenase |
| *PG0980* | hypothetical protein |
| *PG0984* | hypothetical protein |
| *PG0985* | RNA polymerase sigma-70 factor, ECF subfamily |
| *PG0986* | hypothetical protein |
| *PG0987* | hypothetical protein |
| *PG0989* | ribosomal protein L20 |
| *PG0990* | ribosomal protein L35 |
| *PG0991* | translation initiation factor IF-3 |
| *PG0992* | threonyl-tRNA synthetase |
| *PG0995* | hypothetical protein |
| *PG0996* | conserved hypothetical protein TIGR01777 |
| *PG0997* | transcriptional regulator, putative |
| *PG0999* | hypothetical protein |
| *PG1000* | hypothetical protein |
| *PG1001* | conserved hypothetical protein |
| *PG1003* | conserved hypothetical protein |
| *PG1004* | prolyl oligopeptidase family protein |
| *PG1005* | lipoprotein, putative |
| *PG1006* | hypothetical protein |
| *PG1007* | transcriptional regulator, GntR family |
| *PG1008* | hypothetical protein |
| *PG1009* | hypothetical protein |
| *PG1012* | tRNA-i(6)A37 modification enzyme MiaB |
| *PG1013* | acetyl-CoA hydrolase-transferase family protein |
| *PG1017* | pyruvate phosphate dikinase |
| *PG1019* | lipoprotein, putative |
| *PG1020* | hypothetical protein |
| *PG1022* | hypothetical protein |
| *PG1023* | para-aminobenzoate synthase, component I, authentic point mutation |
| *PG1024* | hypothetical protein |
| *PG1027* | hypothetical protein |
| *PG1030* | hypothetical protein |
| *PG1033* | conserved hypothetical protein |
| *PG1034* | ABC transporter, ATP-binding protein |
| *PG1035* | hypothetical protein |
| *PG1036* | excinuclease ABC, A subunit |
| *PG1037* | hypothetical protein |
| *PG1038* | ATP-dependent DNA helicase UvrD-PcrA-Rep Family |
| *PG1039* | integral membrane protein |
| *PG1041* | K+-dependent Na+-Ca+ exchanger related-protein |
| *PG1042* | glycogen synthase, putative |
| *PG1043* | ferrous iron transport protein B |
| *PG1044* | iron dependent repressor, putative |
| *PG1048* | N-acetylmuramoyl-L-alanine amidase, family 3 |
| *PG1049* | conserved hypothetical protein |
| *PG1050* | hypothetical protein |
| *PG1051* | hypothetical protein |
| *PG1052* | transcriptional regulator, putative |
| *PG1053* | transcriptional regulator, putative |
| *PG1056* | conserved hypothetical protein |
| *PG1057* | conserved hypothetical protein |
| *PG1058* | OmpA family protein |
| *PG1060* | carboxyl-terminal protease |
| *PG1064* | dihydroorotate dehydrogenase, putative |
| *PG1065* | dihydroorotate dehydrogenase |
| *PG1066* | butyrate-acetoacetate CoA-transferase, subunit A |
| *PG1067* | conserved hypothetical protein |
| *PG1068* | conserved hypothetical protein |
| *PG1069* | alcohol dehydrogenase, zinc-containing, putative |
| *PG1070* | L-lysine 2,3-aminomutase |
| *PG1071* | conserved hypothetical protein |
| *PG1072* | MutS family protein |
| *PG1073* | D-lysine 5,6-aminomutase, alpha subunit |
| *PG1074* | D-lysine 5,6-aminomutase, beta subunit |
| *PG1075* | coenzyme A transferase, beta subunit |
| *PG1076* | acyl-CoA dehydrogenase, short-chain specific |
| *PG1077* | electron transfer flavoprotein, beta subunit |
| *PG1078* | electron transfer flavoprotein, alpha subunit |
| *PG1079* | enoyl-CoA hydratase-isomerase family protein |
| *PG1080* | 3-hydroxyacyl-CoA dehydrogenase family protein |
| *PG1081* | acetate kinase |
| *PG1082* | phosphotransacetylase |
| *PG1083* | hypothetical protein |
| *PG1084* | thioredoxin family protein |
| *PG1085* | hypothetical protein |
| *PG1087* | radical SAM protein, TIGR01212 family |
| *PG1088* | acetyltransferase, GNAT family |
| *PG1089* | DNA-binding response regulator RprY |
| *PG1091* | DHH subfamily 1 protein |
| *PG1093* | hypothetical protein |
| *PG1094* | phosphomannomutase |
| *PG1095* | RNA methyltransferase, TrmA family |
| *PG1096* | hypothetical protein |
| *PG1097* | Mur ligase domain protein-alanine racemase |
| *PG1098* | hypothetical protein |
| *PG1099* | glucokinase regulator-related protein |
| *PG1100* | hypothetical protein |
| *PG1101* | sodium:solute symporter family protein |
| *PG1103* | ATPase, AAA family |
| *PG1104* | conserved hypothetical protein |
| *PG1105* | RNA polymerase sigma-54 factor |
| *PG1106* | UDP-N-acetylmuramoylalanyl-D-glutamyl-2,6-diaminopimelate--D-alanyl-D-alanyl ligase |
| *PG1114* | aspartate-1-decarboxylase |
| *PG1116* | methylenetetrahydrofolate dehydrogenase-methenyltetrahydrofolate cyclohydrolase |
| *PG1117* | MATE efflux family protein |
| *PG1118* | clpB protein |
| *PG1121* | asparaginyl-tRNA synthetase |
| *PG1122* | ribosomal large subunit pseudouridine synthase B |
| *PG1123* | adenylosuccinate lyase |
| *PG1124* | ATP:cob(I)alamin adenosyltransferase, putative |
| *PG1125* | hypothetical protein |
| *PG1126* | uracil permease |
| *PG1127* | transcriptional regulator, AsnC Family |
| *PG1128* | exodeoxyribonuclease VII, large subunit |
| *PG1129* | ribonucleotide reductase |
| *PG1132* | valyl-tRNA synthetase |
| *PG1133* | hypothetical protein |
| *PG1134* | thioredoxin reductase |
| *PG1135* | bacterial sugar transferase |
| *PG1136* | conserved hypothetical protein |
| *PG1137* | porS protein |
| *PG1138* | pigmentation and extracellular proteinase regulator |
| *PG1139* | hypothetical protein |
| *PG1140* | glycosyl transferase, group 2 family protein |
| *PG1142* | exopolysaccharide synthesis-related protein |
| *PG1144* | peptide chain release factor 2, programmed frameshift |
| *PG1151* | alcohol dehydrogenase, iron-containing |
| *PG1152* | hypothetical protein |
| *PG1153* | hypothetical protein |
| *PG1155* | ADP-heptose--LPS heptosyltransferase, putative |
| *PG1156* | S4 domain protein |
| *PG1159* | cobalamin biosynthesis protein CbiB |
| *PG1160* | L-threonine-O-3-phosphate decarboxylase, putative |
| *PG1161* | cobyric acid synthase CobQ, authentic frameshift |
| *PG1162* | ATP:cob(I)alamin adenosyltransferase, putative |
| *PG1163* | cobyrinic acid a,c-diamide synthase |
| *PG1169* | hypothetical protein |
| *PG1171* | oxidoreductase, putative |
| *PG1172* | iron-sulfur cluster binding protein, putative |
| *PG1173* | YkgG family protein |
| *PG1174* | thioesterase family protein |
| *PG1175* | ABC transporter, ATP-binding protein, putative |
| *PG1176* | ABC transporter, ATP-binding protein, putative |
| *PG1178* | hypothetical protein |
| *PG1179* | conserved hypothetical protein |
| *PG1180* | membrane protein, putative |
| *PG1181* | transcriptional regulator, tetR family |
| *PG1184* | alginate O-acetyltransferase, putative |
| *PG1185* | hypothetical protein |
| *PG1186* | hypothetical protein |
| *PG1189* | hypothetical protein |
| *PG1190* | glycerate dehydrogenase |
| *PG1195* | 8-amino-7-oxononanoate synthase |
| *PG1196* | hypothetical protein |
| *PG1198* | hypothetical protein |
| *PG1208* | dnaK protein |
| *PG1209* | hypothetical protein |
| *PG1210* | peptidase, M24 family |
| *PG1211* | hexapeptide transferase family protein |
| *PG1212* | TPR domain protein |
| *PG1213* | ribonuclease H |
| *PG1214* | hypothetical protein |
| *PG1216* | hypothetical protein |
| *PG1217* | hypothetical protein |
| *PG1218* | hypothetical protein |
| *PG1219* | hypothetical protein |
| *PG1220* | erythronate-4-phosphate dehydrogenase, putative |
| *PG1221* | oxidoreductase, short chain dehydrogenase-reductase family |
| *PG1223* | hypothetical protein |
| *PG1224* | ABC transporter, periplasmic substrate-binding protein, putative, degenerate |
| *PG1225* | ABC transporter, ATP-binding protein |
| *PG1226* | peptidyl-prolyl cis-trans isomerase, cyclophilin-type |
| *PG1229* | hypothetical protein |
| *PG1230* | hypothetical protein |
| *PG1232* | glutamate dehydrogenase, NAD-specific |
| *PG1233* | hypothetical protein |
| *PG1235* | epimerase-reductase, putative |
| *PG1236* | hypothetical protein |
| *PG1237* | transcriptional regulator, LuxR family |
| *PG1238* | ribosomal large subunit pseudouridine synthase family protein |
| *PG1239* | 3-oxoacyl-(acyl-carrier-protein) reductase |
| *PG1241* | GTP-binding protein Lepa |
| *PG1242* | replicative DNA helicase |
| *PG1246* | alanyl-tRNA synthetase |
| *PG1247* | 3-dehydroquinate synthase |
| *PG1248* | conserved hypothetical protein |
| *PG1249* | 1-acyl-sn-glycerol-3-phosphate acetyltransferase, putative |
| *PG1251* | hypothetical protein |
| *PG1252* | membrane protein, putative |
| *PG1253* | DNA ligase, NAD-dependent |
| *PG1254* | acetyltransferase, GNAT family |
| *PG1255* | recombination protein RecR |
| *PG1256* | ribonuclease, Rne-Rng family |
| *PG1258* | DNA-binding protein HU |
| *PG1259* | anaerobic ribonucleoside-triphosphate reductase activating protein |
| *PG1260* | anaerobic ribonucleoside-triphosphate reductase, putative |
| *PG1268* | hypothetical protein |
| *PG1269* | delta-1-pyrroline-5-carboxylate dehydrogenase |
| *PG1270* | conserved hypothetical protein |
| *PG1271* | acetylornithine aminotransferase, putative |
| *PG1273* | hypothetical protein |
| *PG1277* | UDP-glucose-6 dehydrogenase, putative |
| *PG1278* | phosphoserine aminotransferase |
| *PG1279* | D-isomer specific 2-hydroxyacid dehydrogenase family protein |
| *PG1280* | conserved hypothetical protein |
| *PG1281* | hypothetical protein |
| *PG1282* | conserved hypothetical protein |
| *PG1283* | conserved hypothetical protein |
| *PG1285* | glucosamine-6-phosphate isomerase, putative |
| *PG1286* | ferritin |
| *PG1288* | GDP-mannose 4,6-dehydratase |
| *PG1289* | GDP-fucose synthetase |
| *PG1290* | branched-chain amino acid aminotransferase |
| *PG1291* | hypothetical protein |
| *PG1294* | ferrous iron transport protein B |
| *PG1296* | hypothetical protein |
| *PG1297* | ribosomal protein S1 |
| *PG1301* | hypothetical protein |
| *PG1302* | hypothetical protein |
| *PG1303* | helicase, putative |
| *PG1304* | hypothetical protein |
| *PG1305* | glycine cleavage system P protein |
| *PG1306* | metallo-beta-lactamase family protein |
| *PG1307* | glucose-inhibited division protein B |
| *PG1308* | hypothetical protein |
| *PG1310* | exsB protein |
| *PG1311* | conserved hypothetical protein |
| *PG1312* | capA protein, putative |
| *PG1313* | dipeptidase-related protein |
| *PG1314* | chorismate synthase |
| *PG1315* | peptidyl-prolyl cis-trans isomerase SlyD, FKBP-type |
| *PG1316* | hypothetical protein |
| *PG1317* | hypothetical protein |
| *PG1318* | RNA polymerase sigma-70 factor, ECF subfamily |
| *PG1321* | formate--tetrahydrofolate ligase |
| *PG1323* | PhoH family protein |
| *PG1324* | crossover junction endodeoxyribonuclease RuvC |
| *PG1325* | hypothetical protein |
| *PG1327* | aminotransferase, putative |
| *PG1328* | CoA ligase family protein |
| *PG1329* | ISPg5, transposase Orf2, degenerate |
| *PG1330* | large conductance mechanosensitive channel protein |
| *PG1331* | NAD(P) transhydrogenase, alpha subunit, authentic frameshift |
| *PG1333* | hypothetical protein |
| *PG1334* | band 7-Mec-2 family protein |
| *PG1337* | umuD protein |
| *PG1338* | umuC protein |
| *PG1340* | L-lactate permease |
| *PG1341* | hypothetical protein |
| *PG1342* | UDP-N-acetylenolpyruvoylglucosamine reductase |
| *PG1343* | lipoate-protein ligase B |
| *PG1345* | glycosyl transferase, group 1 family protein |
| *PG1346* | glycosyl transferase, group 1 family protein |
| *PG1347* | conserved hypothetical protein |
| *PG1348* | conserved hypothetical protein TIGR00147 |
| *PG1351* | hypothetical protein |
| *PG1352* | hypothetical protein |
| *PG1353* | orotate phosphoribosyltransferase |
| *PG1354* | hydrolase, carbon-nitrogen family |
| *PG1355* | acyltransferase, putative |
| *PG1356* | hypothetical protein |
| *PG1358* | acetyltransferase, GNAT family |
| *PG1359* | hypothetical protein |
| *PG1360* | phosphoribosylamine--glycine ligase |
| *PG1361* | dipeptidyl aminopeptidase IV, putative |
| *PG1362* | conserved hypothetical protein |
| *PG1363* | hypothetical protein |
| *PG1364* | 1-deoxy-D-xylulose 5-phosphate reductoisomerase |
| *PG1365* | 16S rRNA processing protein RimM, putative |
| *PG1366* | UDP-N-acetylglucosamine 1-carboxyvinyltransferase |
| *PG1367* | hypothetical protein |
| *PG1368* | glucose-6-phosphate isomerase |
| *PG1369* | glycerol-3-phosphate dehydrogenase (NAD(P)+) |
| *PG1370* | lysyl-tRNA synthetase |
| *PG1371* | phosphorylase family protein |
| *PG1372* | hypothetical protein |
| *PG1374* | immunoreactive 47 kDa antigen PG97 |
| *PG1375* | hypothetical protein |
| *PG1378* | A-G-specific adenine glycosylase |
| *PG1379* | ABC transporter, periplasmic substrate-binding protein, putative |
| *PG1380* | ABC transporter, ATP-binding protein |
| *PG1381* | ABC transporter, permease protein |
| *PG1382* | hypothetical protein |
| *PG1383* | amino acid exporter, putative |
| *PG1385* | TPR domain protein |
| *PG1386* | DNA gyrase, A subunit |
| *PG1387* | hypothetical protein |
| *PG1388* | hypothetical protein |
| *PG1389* | DNA-binding protein, histone-like family |
| *PG1391* | hypothetical protein |
| *PG1392* | rod shape-determining protein RodA, putative |
| *PG1393* | penicillin-binding protein 2, putative |
| *PG1394* | hypothetical protein |
| *PG1395* | cell shape-determining protein MreC, putative |
| *PG1396* | cell shape-determining protein MreB |
| *PG1397* | phosphoribosylaminoimidazolecarboxamide formyltransferase-IMP cyclohydrolase |
| *PG1401* | beta-eliminating lyase |
| *PG1402* | AP endonuclease domain protein |
| *PG1403* | rhomboid family protein |
| *PG1404* | rhomboid family protein |
| *PG1405* | hypothetical protein |
| *PG1406* | ISPg2, transposase, truncation |
| *PG1407* | nitroimidazole resistance protein, putative |
| *PG1408* | heavy metal efflux pump, CzcD family |
| *PG1409* | hypothetical protein |
| *PG1410* | hypothetical protein |
| *PG1411* | potassium uptake protein TrkA, putative |
| *PG1414* | hypothetical protein |
| *PG1416* | enoyl-(acyl-carrier-protein) reductase II |
| *PG1417* | fumarate hydratase class I, anaerobic |
| *PG1418* | DNA polymerase III, gamma and tau subunits |
| *PG1421* | ferredoxin, 4Fe-4S |
| *PG1422* | D-alanyl-D-alanine carboxypeptidase |
| *PG1423* | hypothetical protein |
| *PG1424* | peptidylarginine deiminase |
| *PG1427* | thiol protease-hemagglutinin PrtT precursor, putative |
| *PG1428* | 6,7-dimethyl-8-ribityllumazine synthase |
| *PG1429* | hypothetical protein |
| *PG1430* | TPR domain protein |
| *PG1431* | DNA-binding response regulator, LuxR family |
| *PG1432* | sensor histidine kinase |
| *PG1433* | hydrolase |
| *PG1434* | 4-diphosphocytidyl-2C-methyl-D-erythritol synthase |
| *PG1536* | cell division protein FtsX, putative |
| *PG1537* | conserved hypothetical protein |
| *PG1538* | undecaprenol kinase, putative |
| *PG1539* | tRNA pseudouridine synthase B |
| *PG1540* | S-adenosylmethionine:tRNA ribosyltransferase-isomerase |
| *PG1541* | 2-amino-4-hydroxy-6-hydroxymethyldihydropteridine pyrophosphokinase |
| *PG1543* | thioesterase family protein |
| *PG1544* | yaaA protein |
| *PG1545* | superoxide dismutase, Fe-Mn |
| *PG1547* | hypothetical protein |
| *PG1548* | thiol protease-hemagglutinin PrtT precursor, authentic frameshift |
| *PG1549* | hypothetical protein |
| *PG1551* | hmuY protein |
| *PG1552* | TonB-dependent receptor HmuR |
| *PG1554* | hypothetical protein |
| *PG1555* | conserved domain protein |
| *PG1556* | conserved hypothetical protein |
| *PG1559* | glycine cleavage system T protein |
| *PG1560* | dTDP-glucose 4,6-dehydratase |
| *PG1561* | dTDP-4-dehydrorhamnose reductase |
| *PG1562* | dTDP-4-dehydrorhamnose 3,5-epimerase |
| *PG1563* | glucose-1-phosphate thymidylyltransferase |
| *PG1564* | membrane protein, putative |
| *PG1565* | 3-deoxy-D-manno-octulosonic-acid transferase, putative |
| *PG1566* | glutamyl-tRNA synthetase |
| *PG1570* | rhodanese-like domain protein |
| *PG1571* | metallo-beta-lactamase superfamily protein |
| *PG1572* | membrane protein, putative |
| *PG1573* | transcriptional regulator, Crp family |
| *PG1576* | L-aspartate oxidase |
| *PG1577* | nicotinate-nucleotide pyrophosphorylase |
| *PG1578* | quinolinate synthetase complex, subunit A |
| *PG1579* | ATPase, MoxR family |
| *PG1580* | conserved hypothetical protein |
| *PG1581* | hypothetical protein |
| *PG1582* | batA protein |
| *PG1583* | batB protein |
| *PG1584* | batC protein |
| *PG1585* | batD protein |
| *PG1586* | batE protein |
| *PG1587* | PAP2 superfamily protein |
| *PG1588* | conserved hypothetical protein |
| *PG1589* | dihydropteroate synthase |
| *PG1592* | HDIG domain protein |
| *PG1593* | shikimate kinase |
| *PG1594* | ComEC-Rec2-related protein |
| *PG1595* | ribulose-phosphate 3-epimerase |
| *PG1596* | isoleucyl-tRNA synthetase, putative |
| *PG1597* | DnaK suppressor protein, putative |
| *PG1598* | lipoprotein signal peptidase, putative |
| *PG1599* | hypothetical protein |
| *PG1600* | membrane protein, putative |
| *PG1601* | biotin--acetyl-CoA-carboxylase ligase |
| *PG1602* | conserved hypothetical protein |
| *PG1603* | HAM1 protein |
| *PG1604* | immunoreactive 84 kDa antigen PG93 |
| *PG1605* | aminopeptidase C |
| *PG1608* | methylmalonyl-CoA decarboxylase, beta subunit |
| *PG1609* | methylmalonyl-CoA decarboxylase, gamma subunit |
| *PG1610* | hypothetical protein |
| *PG1611* | hypothetical protein |
| *PG1612* | methylmalonyl-CoA decarboxylase, alpha subunit |
| *PG1613* | glyoxalase family protein |
| *PG1614* | fumarate reductase, iron-sulfur protein |
| *PG1615* | fumarate reductase, flavoprotein subunit |
| *PG1616* | conserved hypothetical protein |
| *PG1618* | conserved hypothetical protein |
| *PG1619* | biotin synthesis protein BioC, putative |
| *PG1620* | carboxyl-terminal protease-related protein |
| *PG1622* | DNA topoisomerase IV, A subunit, putative |
| *PG1625* | hypothetical protein |
| *PG1626* | hypothetical protein |
| *PG1632* | aldose 1-epimerase |
| *PG1633* | galactokinase |
| *PG1634* | hypothetical protein |
| *PG1635* | hypothetical protein |
| *PG1636* | FtsK-SpoIIIE family protein |
| *PG1638* | thioredoxin family protein |
| *PG1639* | hypothetical protein |
| *PG1640* | DNA-damage-inducible protein F |
| *PG1641* | phosphotyrosine protein phosphatase |
| *PG1647* | cardiolipin synthetase |
| *PG1648* | RelA-SpoT family protein |
| *PG1651* | TPR domain protein |
| *PG1652* | hypothetical protein |
| *PG1654* | D-alanyl-D-alanine dipeptidase |
| *PG1656* | methylmalonyl-CoA mutase, small subunit |
| *PG1657* | methylmalonyl-CoA mutase, large subunit |
| *PG1661* | hypothetical protein |
| *PG1662* | hypothetical protein |
| *PG1663* | ABC transporter, ATP-binding protein |
| *PG1664* | ABC transporter, permease protein, putative |
| *PG1665* | ABC transporter, permease protein, putative |
| *PG1666* | efflux transporter, MFP component, RND family |
| *PG1667* | outer membrane efflux protein |
| *PG1674* | hemagglutinin protein HagB, degenerate |
| *PG1675* | hypothetical protein |
| *PG1676* | phosphoenolpyruvate carboxykinase (ATP) |
| *PG1677* | phosphoglycerate kinase |
| *PG1678* | hypothetical protein |
| *PG1679* | hypothetical protein |
| *PG1680* | ABC transporter, ATP-binding protein, authentic frameshift |
| *PG1681* | glycogen debranching enzyme, archaeal type, putative |
| *PG1682* | glycosyl transferase, group 1 family protein |
| *PG1683* | conserved hypothetical protein |
| *PG1684* | hypothetical protein |
| *PG1687* | HIT family protein |
| *PG1688* | transcription elongation factor GreA |
| *PG1690* | Sua5-YciO-YrdC-YwlC family protein |
| *PG1691* | conserved domain protein |
| *PG1692* | ABC transporter, ATP-binding protein |
| *PG1693* | HesA-MoeB-ThiF family protein |
| *PG1694* | conserved hypothetical protein |
| *PG1695* | hypothetical protein |
| *PG1701* | glutamine amidotransferase, class II-dipeptidase |
| *PG1702* | DNA gyrase, B subunit |
| *PG1703* | MazG family protein |
| *PG1704* | thiol:disulfide interchange protein dsbD, putative |
| *PG1705* | ribosomal large subunit pseudouridine synthase family protein |
| *PG1706* | hypothetical protein |
| *PG1707* | hypothetical protein |
| *PG1712* | alpha-1,2-mannosidase family protein |
| *PG1713* | lipoprotein, putative |
| *PG1714* | pyridoxamine-phosphate oxidase |
| *PG1715* | hypothetical protein |
| *PG1718* | hypothetical protein |
| *PG1719* | ABC transporter, ATP-binding protein, MsbA family |
| *PG1720* | conserved domain protein |
| *PG1721* | ribonuclease R |
| *PG1722* | hypothetical protein |
| *PG1723* | ribosomal protein S20 |
| *PG1724* | O-sialoglycoprotein endopeptidase |
| *PG1725* | competence-damage-inducible protein CinA domain protein |
| *PG1726* | PDZ domain protein |
| *PG1727* | yitL protein |
| *PG1728* | cytidine-deoxycytidylate deaminase family protein |
| *PG1729* | thiol peroxidase |
| *PG1730* | O-methyltransferase family protein |
| *PG1731* | 3-dehydroquinate dehydratase, type II |
| *PG1732* | integrase-recombinase XerD |
| *PG1733* | hypothetical protein |
| *PG1734* | transporter, putative |
| *PG1739* | conserved domain protein |
| *PG1741* | aspartate ammonia-lyase |
| *PG1743* | 2-dehydro-3-deoxyphosphooctonate aldolase |
| *PG1745* | phosphoribulokinase family protein |
| *PG1747* | ribose 5-phosphate isomerase B, putative |
| *PG1748* | transketolase |
| *PG1750* | alpha-1,3-4-fucosidase, putative |
| *PG1751* | aminotransferase, class V |
| *PG1752* | hypothetical protein |
| *PG1753* | selenide, water dikinase |
| *PG1754* | conserved domain protein |
| *PG1755* | fructose-bisphosphate aldolase, class I |
| *PG1758* | ribosomal protein S15 |
| *PG1760* | ABC transporter, ATP-binding protein |
| *PG1761* | acetyltransferase, GNAT family |
| *PG1762* | protein-export membrane protein SecD-protein-export membrane protein SecF |
| *PG1763* | ribonuclease III |
| *PG1764* | 3-oxoacyl-(acyl-carrier-protein) synthase II |
| *PG1765* | acyl carrier protein |
| *PG1766* | phosphoribosylglycinamide formyltransferase |
| *PG1767* | lipoprotein, putative |
| *PG1768* | magnesium chelatase, subunit D-I family |
| *PG1769* | hypothetical protein |
| *PG1770* | hypothetical protein |
| *PG1771* | phenylalanyl-tRNA synthetase, alpha subunit |
| *PG1772* | endonuclease III |
| *PG1773* | PAP2 superfamily protein |
| *PG1774* | transcription-repair coupling factor |
| *PG1775* | grpE protein |
| *PG1776* | dnaJ protein |
| *PG1778* | conserved hypothetical protein |
| *PG1779* | conserved hypothetical protein |
| *PG1780* | 8-amino-7-oxononanoate synthase |
| *PG1781* | uridine kinase |
| *PG1782* | hypothetical protein |
| *PG1783* | glycosyl transferase, group 2 family protein |
| *PG1784* | conserved hypothetical protein |
| *PG1787* | hypothetical protein |
| *PG1788* | cysteine peptidase, putative |
| *PG1789* | peptidyl-dipeptidase Dcp |
| *PG1790* | hypothetical protein |
| *PG1791* | hypothetical protein |
| *PG1792* | sodium-hydrogen antiporter |
| *PG1793* | 1,4-alpha-glucan branching enzyme |
| *PG1794* | DNA polymerase type I |
| *PG1795* | hypothetical protein |
| *PG1797* | DNA-binding response regulator-sensor histidine kinase |
| *PG1798* | immunoreactive 46 kDa antigen PG99 |
| *PG1799* | hypothetical protein |
| *PG1801* | v-type ATPase, subunit E, putative |
| *PG1802* | hypothetical protein |
| *PG1803* | v-type ATPase, subunit A |
| *PG1804* | v-type ATPase, subunit B |
| *PG1805* | v-type ATPase, subunit D |
| *PG1806* | v-type ATPase, subunit I |
| *PG1807* | v-type ATPase, subunit K |
| *PG1808* | guanosine-3,5-bis(diphosphate) 3-pyrophosphohydrolase |
| *PG1809* | 2-oxoglutarate oxidoreductase, gamma subunit |
| *PG1810* | 2-oxoglutarate oxidoreductase, beta subunit |
| *PG1811* | hypothetical protein |
| *PG1812* | 2-oxoglutarate oxidoreductase, alpha subunit |
| *PG1813* | ferredoxin, 4Fe-4S |
| *PG1815* | 3-deoxy-D-manno-octulosonate cytidylyltransferase |
| *PG1816* | NAD(P)H dehydrogenase, quinone family, putative |
| *PG1817* | conserved hypothetical protein |
| *PG1818* | hypothetical protein |
| *PG1819* | hypothetical protein |
| *PG1820* | cytochrome c nitrite reductase, catalytic subunit NrfA |
| *PG1821* | cytochrome c nitrite reductase, small subunit NrfH |
| *PG1823* | hypothetical protein |
| *PG1824* | enolase |
| *PG1825* | hypothetical protein |
| *PG1826* | conserved domain protein |
| *PG1827* | RNA polymerase sigma-70 factor, ECF subfamily |
| *PG1828* | lipoprotein, putative |
| *PG1829* | long-chain-fatty-acid--CoA ligase, putative |
| *PG1831* | ATP-dependent DNA helicase RecQ |
| *PG1834* | glycogen synthase-related protein |
| *PG1835* | lipoprotein, putative |
| *PG1836* | nucleoside permease NupG |
| *PG1837* | hemagglutinin protein HagA |
| *PG1840* | conserved domain protein |
| *PG1841* | conserved hypothetical protein |
| *PG1842* | acetyltransferase, GNAT family |
| *PG1844* | hemagglutinin protein HagD |
| *PG1847* | endoribonuclease L-PSP, putative |
| *PG1848* | RNA methyltransferase, TrmH family |
| *PG1849* | DNA repair protein RecN |
| *PG1850* | hypothetical protein |
| *PG1851* | phosphopantothenoylcysteine decarboxylase-phosphopantothenate--cysteine ligase |
| *PG1852* | exonuclease |
| *PG1853* | DNA polymerase III, beta subunit |
| *PG1854* | 5-formyltetrahydrofolate cyclo-ligase family protein |
| *PG1855* | carboxyl-terminal protease |
| *PG1856* | cytidine-deoxycytidylate deaminase family protein |
| *PG1857* | conserved hypothetical protein |
| *PG1858* | flavodoxin |
| *PG1859* | glycerate kinase family protein |
| *PG1860* | conserved hypothetical protein |
| *PG1861* | hypothetical protein |
| *PG1862* | hypothetical protein |
| *PG1863* | hypothetical protein |
| *PG1868* | membrane protein, putative |
| *PG1871* | hypothetical protein |
| *PG1874* | conserved hypothetical protein |
| *PG1875* | hemolysin |
| *PG1876* | conserved hypothetical protein |
| *PG1878* | cysteinyl-tRNA synthetase |
| *PG1879* | conserved hypothetical protein |
| *PG1880* | glycosyl transferase, group 2 family protein |
| *PG1881* | hypothetical protein |
| *PG1884* | alpha-L-fucosidase precursor, putative |
| *PG1885* | polyphosphate kinase |
| *PG1886* | GTP-binding protein HflX |
| *PG1887* | rhodanese-like domain protein |
| *PG1895* | hypothetical protein |
| *PG1896* | S-adenosylmethionine synthase |
| *PG1897* | thiamine pyrophosphokinase |
| *PG1898* | transporter, putative |
| *PG1899* | TonB-dependent receptor, putative |
| *PG1900* | conserved hypothetical protein TIGR00157 |
| *PG1901* | ribosome recycling factor |
| *PG1902* | uridylate kinase |
| *PG1903* | conserved hypothetical protein |
| *PG1904* | hypothetical protein |
| *PG1910* | ribosomal protein L17 |
| *PG1911* | DNA-directed RNA polymerase, alpha subunit |
| *PG1912* | ribosomal protein S4 |
| *PG1913* | ribosomal protein S11 |
| *PG1914* | ribosomal protein S13 |
| *PG1915* | ribosomal protein L36 |
| *PG1916* | translation initiation factor IF-1 |
| *PG1917* | methionine aminopeptidase, type I |
| *PG1918* | preprotein translocase, SecY subunit |
| *PG1919* | ribosomal protein L15 |
| *PG1920* | ribosomal protein L30 |
| *PG1921* | ribosomal protein S5 |
| *PG1922* | ribosomal protein L18 |
| *PG1923* | ribosomal protein L6 |
| *PG1924* | ribosomal protein S8 |
| *PG1925* | ribosomal protein S14 |
| *PG1926* | ribosomal protein L5 |
| *PG1927* | ribosomal protein L24 |
| *PG1928* | ribosomal protein L14 |
| *PG1929* | ribosomal protein S17 |
| *PG1930* | ribosomal protein L29 |
| *PG1931* | ribosomal protein L16 |
| *PG1932* | ribosomal protein S3 |
| *PG1933* | ribosomal protein L22 |
| *PG1934* | ribosomal protein S19 |
| *PG1935* | ribosomal protein L2 |
| *PG1936* | ribosomal protein L23 |
| *PG1937* | ribosomal protein L4 |
| *PG1938* | ribosomal protein L3 |
| *PG1939* | ribosomal protein S10 |
| *PG1940* | translation elongation factor G |
| *PG1941* | ribosomal protein S7 |
| *PG1942* | ribosomal protein S12 |
| *PG1943* | hypothetical protein |
| *PG1944* | 3-phosphoshikimate 1-carboxyvinyltransferase |
| *PG1945* | hypothetical protein |
| *PG1946* | ABC 3 transporter family protein |
| *PG1947* | TPR domain protein |
| *PG1948* | lipoprotein, putative |
| *PG1949* | malate dehydrogenase |
| *PG1950* | membrane protein, putative |
| *PG1951* | glutaminyl-tRNA synthetase |
| *PG1952* | DedA family protein |
| *PG1953* | YitT family protein |
| *PG1954* | NAD dependent epimerase-reductase-related protein |
| *PG1956* | 4-hydroxybutyrate CoA-transferase |
| *PG1959* | ribosomal protein L33 |
| *PG1960* | ribosomal protein L28 |
| *PG1961* | hypothetical protein |
| *PG1963* | Sua5-YciO-YrdC-YwlC family protein |
| *PG1964* | bacterial sugar transferase |
| *PG1965* | voltage gated chloride channel, authentic frameshift |
| *PG1966* | conserved hypothetical protein |
| *PG1967* | TPR domain protein |
| *PG1992* | glucose-inhibited division protein A |
| *PG1993* | excinuclease ABC, C subunit |
| *PG1994* | D-tyrosyl-tRNA(Tyr) deacylase |
| *PG1995* | conserved hypothetical protein |
| *PG1996* | deoxyribose-phosphate aldolase |
| *PG1997* | hypothetical protein |
| *PG1998* | polyprenyl synthetase |
| *PG1999* | conserved hypothetical protein |
| *PG2000* | signal peptidase-related protein |
| *PG2001* | signal peptidase I |
| *PG2002* | dihydrodipicolinate reductase |
| *PG2003* | deoxyguanosinetriphosphate triphosphohydrolase |
| *PG2004* | conserved hypothetical protein |
| *PG2006* | hypothetical protein |
| *PG2008* | TonB-dependent receptor, putative |
| *PG2009* | DNA repair protein RecO, putative |
| *PG2010* | phosphomannomutase, putative |
| *PG2013* | CRISPR-associated protein Cas2 |
| *PG2014* | CRISPR-associated protein Cas1 |
| *PG2015* | CRISPR-associated protein Cas4 |
| *PG2020* | CRISPR-associated protein, TM1814 family |
| *PG2021* | conserved hypothetical protein |
| *PG2022* | hypothetical protein |
| *PG2023* | methionyl-tRNA formyltransferase |
| *PG2026* | phosphoglycerate mutase family protein |
| *PG2027* | hypothetical protein |
| *PG2028* | ebsC protein |
| *PG2029* | hypothetical protein |
| *PG2030* | hypothetical protein |
| *PG2031* | hypothetical protein |
| *PG2032* | primosomal protein N |
| *PG2033* | glutamate synthase, small subunit |
| *PG2034* | oxidoreductase, FAD-binding, putative |
| *PG2035* | tRNA (guanine-N1)-methyltransferase |
| *PG2036* | ion transporter |
| *PG2037* | hypothetical protein |
| *PG2038* | N-acetylmuramoyl-L-alanine amidase, putative |
| *PG2040* | DNA-binding protein, histone-like family |
| *PG2041* | hypothetical protein |
| *PG2043* | conserved hypothetical protein TIGR00486 |
| *PG2044* | conserved hypothetical protein |
| *PG2046* | tRNA(Ile)-lysidine synthetase |
| *PG2047* | helicase, putative |
| *PG2048* | hypothetical protein |
| *PG2049* | hypothetical protein |
| *PG2050* | hypothetical protein |
| *PG2052* | dihydrodipicolinate synthase |
| *PG2053* | dethiobiotin synthase |
| *PG2054* | lipoprotein PG3 |
| *PG2055* | dihydroorotate dehydrogenase family protein |
| *PG2056* | transposase, ISPg2-related, truncation |
| *PG2060* | thymidylate synthase |
| *PG2061* | dihydrofolate reductase |
| *PG2062* | histidyl-tRNA synthetase |
| *PG2064* | hypothetical protein |
| *PG2065* | conserved hypothetical protein TIGR00048 |
| *PG2066* | lipoprotein, putative |
| *PG2067* | pyridoxal phosphate biosynthetic protein PdxA |
| *PG2068* | glycerol-3-phosphate cytidylyltransferase |
| *PG2069* | oxidoreductase, short chain dehydrogenase-reductase family |
| *PG2070* | hypothetical protein |
| *PG2071* | conserved domain protein |
| *PG2072* | UvrD-REP helicase domain protein |
| *PG2078* | conserved hypothetical protein |
| *PG2079* | hypothetical protein |
| *PG2080* | adenosylmethionine--8-amino-7-oxononanoate aminotransferase |
| *PG2081* | biotin synthetase |
| *PG2083* | hypothetical protein |
| *PG2085* | tryptophanyl-tRNA synthetase |
| *PG2086* | hypothetical protein |
| *PG2088* | peptide methionine sulfoxide reductase |
| *PG2089* | hypothetical protein |
| *PG2090* | cation efflux family protein |
| *PG2092* | hypothetical protein |
| *PG2094* | conserved domain protein |
| *PG2095* | lipoprotein, putative |
| *PG2096* | conserved domain protein |
| *PG2097* | ribose-phosphate pyrophosphokinase |
| *PG2099* | ATP-dependent RNA helicase, DEAD-DEAH box family |
| *PG2101* | hypothetical protein |
| *PG2102* | immunoreactive 61 kDa antigen PG91 |
| *PG2103* | hypothetical protein |
| *PG2104* | hypothetical protein |
| *PG2105* | lipoprotein, putative |
| *PG2106* | hypothetical protein |
| *PG2107* | thiH protein |
| *PG2108* | thiG protein |
| *PG2110* | thiamine biosynthesis protein ThiC |
| *PG2111* | thiamine biosynthesis protein ThiS |
| *PG2116* | hypothetical protein |
| *PG2117* | ribosomal protein S16 |
| *PG2119* | oxidoreductase, Gfo-Idh-MocA family |
| *PG2120* | metallo-beta-lactamase superfamily protein |
| *PG2121* | L-asparaginase |
| *PG2122* | ISPg2, transposase, truncation |
| *PG2123* | hypothetical protein |
| *PG2124* | glyceraldehyde 3-phosphate dehydrogenase, type I |
| *PG2125* | transcriptional regulator, AraC family |
| *PG2126* | conserved hypothetical protein TIGR00044 |
| *PG2127* | hypothetical protein |
| *PG2130* | hypothetical protein |
| *PG2131* | 60 kDa protein |
| *PG2133* | lipoprotein, putative |
| *PG2139* | hypothetical protein |
| *PG2140* | ribosomal protein L32 |
| *PG2141* | 3-oxoacyl-(acyl-carrier-protein) synthase III |
| *PG2142* | GTP-binding protein Era |
| *PG2143* | GTP-binding protein, Era-ThdF family |
| *PG2144* | hypothetical protein |
| *PG2145* | polysaccharide deacetylase |
| *PG2146* | conserved hypothetical protein |
| *PG2147* | xanthine phosphoribosyltransferase |
| *PG2148* | xanthine-uracil permease family protein |
| *PG2149* | hypothetical protein |
| *PG2150* | LysM domain protein |
| *PG2153* | transposase, truncation |
| *PG2154* | hypothetical protein |
| *PG2155* | lipoprotein, putative |
| *PG2156* | conserved hypothetical protein TIGR00046 |
| *PG2157* | glutamine cyclotransferase-related protein |
| *PG2158* | conserved hypothetical protein |
| *PG2159* | protoporphyrinogen oxidase |
| *PG2161* | transcriptional regulator, AraC family |
| *PG2162* | lipid A disaccharide synthase |
| *PG2163* | stationary-phase survival protein SurE |
| *PG2164* | peptidyl-prolyl cis-trans isomerase, FKBP-type |
| *PG2165* | glycyl-tRNA synthetase |
| *PG2167* | immunoreactive 53 kDa antigen PG123 |
| *PG2168* | hypothetical protein |
| *PG2170* | sugar transporter |
| *PG2171* | D-isomer specific 2-hydroxyacid dehydrogenase family protein |
| *PG2173* | outer membrane lipoprotein Omp28 |
| *PG2174* | hypothetical protein |
| *PG2175* | conserved hypothetical protein |
| *PG2177* | NADH:ubiquinone oxidoreductase, Na translocating, F subunit |
| *PG2178* | NADH:ubiquinone oxidoreductase, Na translocating, E subunit |
| *PG2179* | NADH:ubiquinone oxidoreductase, Na(+)-translocating, D subunit |
| *PG2180* | NADH:ubiquinone oxidoreductase, Na translocating, C subunit |
| *PG2181* | NADH:ubiquinone oxidoreductase, Na translocating, B subunit |
| *PG2186* | transcriptional regulator, putative |
| *PG2187* | 1,4-dihydroxy-2-naphthoate octaprenyltransferase |
| *PG2188* | diaminopimelate decarboxylase |
| *PG2189* | aspartate kinase |
| *PG2190* | cell-division ATP-binding protein |
| *PG2192* | peptidase, M23-M37 family |
| *PG2195* | ISPg1, transposase, truncation |
| *PG2197* | conserved hypothetical protein |
| *PG2198* | hemagglutinin protein, truncation |
| *PG2199* | ABC transporter, ATP-binding protein, putative |
| *PG2200* | TPR domain protein |
| *PG2201* | peptide deformylase |
| *PG2202* | conserved hypothetical protein TIGR00250 |
| *PG2204* | hypothetical protein |
| *PG2205* | 2-dehydropantoate 2-reductase |
| *PG2206* | ABC transporter, ATP-binding protein |
| *PG2207* | conserved domain protein |
| *PG2210* | excinuclease ABC, A subunit |
| *PG2212* | hypothetical protein |
| *PG2213* | nitrite reductase-related protein |
| *PG2214* | hypothetical protein |
| *PG2215* | mannose-1-phosphate guanylyltransferase |
| *PG2216* | hypothetical protein |
| *PG2217* | deoxyxylulose-5-phosphate synthase |
| *PG2218* | potassium uptake protein TrkA |
| *PG2219* | potassium uptake protein TrkH |
| *PG2220* | hypothetical protein |
| *PG2221* | MiaB-like tRNA modifying enzyme |
| *PG2223* | glycosyl transferase, group 2 family protein |
| *PG2224* | membrane protein, putative |
| *PG2225* | conserved hypothetical protein |
| *PG2226* | hypothetical protein |
| *PG2227* | hypothetical protein |
| **Non aberrant probes called absent** | |
| *PG0662* | hypothetical protein |
| *PG1546* | hypothetical protein |
