# Supplementary material for: The core genome of the anaerobic oral pathogenic bacterium Porphyromonas gingivalis
Source: BMC Microbiol. 2010 Sep 29;10:252. doi: 10.1186/1471-2180-10-252 (PMC2955634; doi:10.1186/1471-2180-10-252)
Supplement: Additional file 2 — W83-specific genes 65 genes. aberrant in each test strain of which 39 W83-specific genes (marked in red) [file 1471-2180-10-252-S2.DOC]

### Additional file 2

Title**:** 65 genes aberrant in each test strain of which 39 W83-specific genes (marked in red)

| **GeneID** | **Annotated function** |
| --- | --- |
| *PG0115* | Hexapeptide transferase family protein |
| *PG0117* | polysaccharide transport protein, putative |
| *PG0118* | glycosyl transferase, group 2 family protein |
| *PG0219* | conserved domain protein |
| *PG0340* | Hypothetical protein |
| *PG0382* | conserved hypothetical protein |
| *PG0456* | PHP N-terminal domain protein |
| *PG0457* | Hypothetical protein |
| *PG0461* | ISPg7, transposase |
| *PG0556* | Hypothetical protein |
| *PG0609* | Hypothetical protein |
| *PG0614* | Hypothetical protein |
| *PG0741* | conserved hypothetical protein |
| *PG0742* | antigen PgaA |
| *PG0834* | Hypothetical protein |
| *PG0838* | Integrase |
| *PG0839* | conserved hypothetical protein |
| *PG0840* | Hypothetical protein |
| *PG0841* | mobilizable transposon, excision protein, putative |
| *PG0842* | mobilizable transposon, hypothetical protein, putative |
| *PG0843* | Hypothetical protein |
| *PG0844* | Hypothetical protein |
| *PG0856* | Hypothetical protein |
| *PG0861* | helicase, SNF2-RAD54 family |
| *PG0862* | type IIS restriction endonuclease, putative |
| *PG0863* | type IIS DNA modification methyltransferase, truncation |
| *PG0864* | site-specific recombinase, resolvase family |
| *PG0866* | Hypothetical protein |
| *PG0875* | mobilizable transposon, tnpA protein |
| *PG0971* | McrBC restriction endonuclease system, McrB subunit, putative |
| *PG0972* | conserved hypothetical protein |
| *PG1059* | Hypothetical protein |
| *PG1107* | Hypothetical protein |
| *PG1108* | Hypothetical protein |
| *PG1109* | Mobilization protein |
| *PG1110* | Hypothetical protein |
| *PG1111* | conserved hypothetical protein, authentic point mutation |
| *PG1112* | Hypothetical protein |
| *PG1113* | Integrase |
| *PG1130* | TPR domain protein |
| *PG1202* | Hypothetical protein |
| *PG1203* | transcriptional regulator, putative |
| *PG1436* | ATPase, putative |
| *PG1439* | Hypothetical protein |
| *PG1440* | Hypothetical protein |
| *PG1442* | Hypothetical protein |
| *PG1511* | Hypothetical protein |
| *PG1512* | conserved domain protein |
| *PG1513* | phosphoribosyltransferase, putative-phosphoglycerate mutase family protein |
| *PG1514* | glycerol dehydrogenase-related protein |
| *PG1515* | ribulose bisphosphate carboxylase-related protein |
| *PG1516* | Hypothetical protein |
| *PG1517* | Transposase, degenerate |
| *PG1518* | ISPg8, transposase, degenerate |
| *PG1519* | hypothetical protein |
| *PG1591* | conserved hypothetical protein |
| *PG1649* | hypothetical protein |
| *PG1864* | leucine-rich protein |
| *PG1894* | hypothetical protein |
| *PG2109* | DNA repair protein RecO, putative |
| *PG2115* | protease PrtT, degenerate |
| *PG2132* | fimbrilin FimA |
| *PG2134* | lipoprotein, putative |
| *PG2135* | lipoprotein, putative |
| *PG2136* | hypothetical protein |
|  |  |
|  |  |
